# Genome-Wide Analysis of Nutrient Signaling Pathways Conserved in Arbuscular Mycorrhizal Fungi

**DOI:** 10.3390/microorganisms9081557

**Published:** 2021-07-22

**Authors:** Xiaoqin Zhou, Jiangyong Li, Nianwu Tang, Hongyun Xie, Xiaoning Fan, Hui Chen, Ming Tang, Xianan Xie

**Affiliations:** 1 State Key Laboratory of Conservation and Utilization of Subtropical Agro-Bioresources, Lingnan Guangdong Laboratory of Modern Agriculture, Guangdong Key Laboratory for Innovative Development and Utilization of Forest Plant Germplasm, College of Forestry and Landscape Architecture, South China Agricultural University, Guangzhou 510642, China; zhouxiaoqin1@stu.scau.edu.cn (X.Z.); hongyunxie@stu.scau.edu.cn (H.X.); fxnxxa@126.com (X.F.); chenhuiyl@163.com (H.C.); 2 Institute for Environmental and Climate Research, Jinan University, Guangzhou 511443, China; yzamm123@163.com; 3 UMR Interactions Arbres/Microorganismes, Centre INRA-Grand Est-Nancy, 54280 Champenoux, France; tangnianwu@163.com

**Keywords:** arbuscular mycorrhizal fungi, nutrient signaling pathways, cAMP/PKA, TOR, SNF1, PHO, appressorium formation, arbuscule longevity

## Abstract

Arbuscular mycorrhizal (AM) fungi form a mutualistic symbiosis with a majority of terrestrial vascular plants. To achieve an efficient nutrient trade with their hosts, AM fungi sense external and internal nutrients, and integrate different hierarchic regulations to optimize nutrient acquisition and homeostasis during mycorrhization. However, the underlying molecular networks in AM fungi orchestrating the nutrient sensing and signaling remain elusive. Based on homology search, we here found that at least 72 gene components involved in four nutrient sensing and signaling pathways, including cAMP-dependent protein kinase A (cAMP-PKA), sucrose non-fermenting 1 (SNF1) protein kinase, target of rapamycin kinase (TOR) and phosphate (PHO) signaling cascades, are well conserved in AM fungi. Based on the knowledge known in model yeast and filamentous fungi, we outlined the possible gene networks functioning in AM fungi. These pathways may regulate the expression of downstream genes involved in nutrient transport, lipid metabolism, trehalase activity, stress resistance and autophagy. The RNA-seq analysis and qRT-PCR results of some core genes further indicate that these pathways may play important roles in spore germination, appressorium formation, arbuscule longevity and sporulation of AM fungi. We hope to inspire further studies on the roles of these candidate genes involved in these nutrient sensing and signaling pathways in AM fungi and AM symbiosis.

## 1. Introduction

Arbuscular mycorrhizal (AM) fungi establish mutualistic associations with approximately 72% of terrestrial vascular plants, obtaining fatty acids and sugars from the host plants in order to complete their obligate life cycle [1,2,3]. As exchange, they provide their host plants with mineral nutrients (e.g., phosphate and nitrogen) [4,5]. AM fungi have evolved the sophisticated mechanisms for the nutrient uptake, transport and metabolism during symbiosis [6,7,8,9]. For example, the AM fungal transporters of monosaccharide, ammonium, nitrate, amino acids and phosphate have been identified [5,10,11,12,13,14,15,16].

The external phosphate and inorganic nitrogen taken up by their membrane-localized transporters are respectively converted into polyphosphate and arginine, to be transported from extraradical mycelium to intraradical mycelium and then delivered to the host plants across the symbiotic interface [17,18,19,20]. Accordingly, AM fungi perceive and utilize these nutrients both from the external environments and the host root cells, in order to facilitate the various cellular processes and regulate the fungal proliferation within roots. Both AM fungi and host plants undergo cellular reprogramming to perceive and interact with each other through the complex and multi-layered signaling networks in which nutrients could act as important players [21,22]. However, most studies have focused on the roles of host plant genes in nutrient exchange and signaling, whereas our knowledge about how the AM fungal genes involved in such processes remains limited [22,23,24].

The nutrient sensing and signaling pathways can respond to multiple nutrients and regulate various aspects throughout the life cycle of the eukaryotic cells, from yeasts to mammals [25,26,27]. The cAMP-dependent Protein Kinase A (cAMP-PKA), Sucrose Non-Fermenting 1 protein kinase (SNF1), Target of Rapamycin kinase (TOR) and Phosphate (PHO) signaling pathways, which are engaged in the carbon, nitrogen and phosphate sensing and signaling, have been studied in *Saccharomyces cerevisiae* and some fungal pathogens [27,28,29,30,31,32]. In our previous study, we found that phosphate treatment or the knockdown of a phosphate transceptor GigmPT affected the expression levels of many genes involved in the PHO, PKA and TOR pathways in AM fungus *G. margarita* [14]. Nevertheless, the interactions among the key components and more downstream targets in AM fungi should be further explored.

In recent years, the genomes of eight AM fungi from the subphylum Glomeromycotina in the Mucoromycota have been released. They are four members of Glomerales, *Rhizophagus irregularis*, *R. clarus*, *R. cerebriforme* and *R. diaphanous*, three members of the Diversisporales: *Diversispora epigea*, *Gigaspora rosea* and *G. margarita*, as well as one member of Archaeosporales, *Geosiphon pyriformis*, which is capable of forming endosymbiosis with nitrogen-fixing cyanobacteria representing the ancestral AM fungus [33,34,35,36,37,38,39]. These genome data will inspire further studies about AM fungal nutrient signaling.

In this study, we found that 72 components involved in the four nutrients sensing and signaling pathways (cAMP/PKA, SNF1, TOR and PHO) are evolutionarily conserved in the six well sequenced genomes of AM fungi. These components of AM fungi share high similarities in amino acid sequences and structural domains with those of *S. cerevisiae* and other twenty fungal species. Moreover, the RNA-sequencing profiles and gene expression patterns detected by qRT-PCR indicate that the key genes in these pathways may play important roles in the life cycle of AM fungi. Based on the knowledge known in yeasts and filamentous fungi, we propose that it is under the regulation of the cAMP-PKA, SNF1, TOR and PHO pathways that AM fungi respond to various available nutrients (e.g., carbon, nitrogen and phosphate) and regulate the expression of the target genes involved in a series of cellular processes, including nutrient transport, lipid metabolism, trehalase activity, stress resistance and autophagy. Furthermore, these pathways may play important roles in the spore germination, appressorium formation, arbuscule longevity and sporulation of AM fungi. This study aimed to throw light upon the nutrient response and homeostasis of AM fungi and provide new insights into the cellular and molecular bases of AM fungi–plant interactions.

## 2. Materials and Methods

### 2.1. Genomic Sequences Searching and Analyses

To identify the candidate genes involved in nutrient sensing and signaling pathways in AM fungi, amino acid sequences of reference genes involved in the cAMP-PKA, SNF1, TOR and PHO signaling pathways in *S. cerevisiae* [27,28] were used as queries to search for homologs in the six available genomes of AM fungi (Table 1). Multiple tBLASTn and BLASTp searches against the selected genomes were performed via genome BLAST at the NCBI database. The best matching sequences that ranked the first place were downloaded for analysis. The identities were generated by BLASTp results. All hits with identities greater than 20% and E-values below 10^−4^ were kept. The conserved domains of amino acid sequences were analyzed by searching the NCBI Conserved Domain Database.

### 2.2. Transcriptome Analysis

To analyze the expression profiles of target genes in different tissues of AM fungi, the original RNA-seq reads of both asymbiotic (germinating spores) and symbiotic tissues (mycorrhizal roots) of *R. irregularis* and *G. rosea* were downloaded from the NCBI GEO database and mapped to their genomes using CLC Genomics Workbench (v20), setting the mapping similarity at 0.95 and fraction at 0.90. Gene expression (FPKM) calculation and differentially expressed gene analysis (FDR-corrected *p* < 0.05, Benjamini–Hochberg test) were also performed using CLC software (v20), as described in Morin et al. (2019) [36]. The GEO accession numbers of RNA-seq reads are as follows: germinating spores (GSM1658278, GSM1658280, GSM1658282) and mycorrhizal roots (GSM1658563, GSM1658564, GSM1658565) of *R. irregularis*, and germinating spores (GSM1657757, GSM1657758, GSM1657759) and mycorrhizal roots (GSM1657861, GSM1657862, GSM1657863) of *G. rosea*.

### 2.3. AM Fungal Materials and Mycorrhization

The spores of *R. irregularis* (DAOM 197198), *G. margarita* (BEG34), *Funneliformis mosseae* (BEG12) and *G. rosea* (Nicolson & Schenck DAOM194757) were used for the mycorrhization experiments. *R. irregularis* or *F. mosseae* was propagated on *Trifolium repens* L. grown in sterilized sands and treated with modified Long Ashton (mLA) solution containing 30 μM KH_2_PO_4_ for 3 months [60]. The seeds of *Astragalus sinicus* [61] were surface-sterilized and incubated for 10 days at 25 °C and then incubated with new spores and root segments of *R. irregularis* (about 100 spores/plant) in the pots and grown at 25/18 °C with a 16 h light/8 h darkness period. The mycorrhizal plants were supplemented once a week with mLA solution containing 30 μM KH_2_PO_4_ [60] and then cultivated for 14, 21, 28, 35, 42 and 56 days after inoculation. Spores of *G. margarita* were collected by wet sieving. The surface-sterilized spores of *F. mosseae* and *G. margarita* were germinated in sterile water containing 10^−8^ or 10^−9^ mol/L GR24 (10^−9^ mol/L GR24 for *G. margarita*, GR24 was dissolved in acetone solution) at 25 °C in a dark incubator for 7 days [14,62]. Fourteen-day-old roots of *A. sinicus* were inoculated with *G. margarita* to establish the *G. margarita*/*A. sinicus* association, while the seedlings of *Solanum lycopersicum* (cv. MoneyMaker) were inoculated with *F. mosseae* by mixing the inoculum with sterile quartz sands (30% *v*/*v*). Both *A. sinicus* and *S. lycopersicum* plants were weekly watered with the mLA solution containing a low (30 μM/L NaH_2_PO_4_) phosphate and harvested 64 days post-inoculation.

### 2.4. Gene Expression Detected by qRT-PCR

Mycorrhizal roots were stored at −80 °C and used for total RNA extraction using a Plant RNA Kit (OMEGA Bio-Tek, Norcross, GA, USA), according to the manufacturer’s instructions. The first strand of cDNA was synthesized from 1 μg of total RNA using a HiScript^®^ III RT SuperMix for qPCR (+gDNA wiper) kit (Vazyme, Nanjing, China), following the manufacturer’s instructions. Real-time qRT-PCR reactions were performed using the ChamQ Univeral SYBR qPCR Master Mix (Vazyme, Nanjing, China), according to the manufacturer’s instructions, and conducted in a 96-well CFX Connect™ Real-time PCR Detection System (Bio-Rad, Hercules, CA, USA). All the reactions were determined in three biological replicates with two technical replicates. The *RiEF1α* was used as the reference gene for normalization of AM fungal genes expression, whereas *AsActin* was used as the reference gene for normalization of *A. sinicus* gene *AsPT4* expression. Relative expression levels of the genes were computed by the 2^−ΔΔCt^ method of relative quantification. The software SPSS 22 (Spss Inc., Chicago, IL, USA) was used to perform statistical analyses. All data of gene expression are shown as the means ± SD. The different letters indicate significant differences (*n* = 3, *p* < 0.05, Duncan’s test). The gene-specific primer sequences are provided in Supplemental Table S3.

### 2.5. Staining Procedures

The tomato roots with *F. mosseae* were stained with 0.01% cotton blue (*w*/*v*) in lactic acid. The *A. sinicus* roots with *G. margarita* or *R. irregularis* were immersed in a 10% KOH (*w*/*v*) solution at 37 °C for one week, neutralized in 2% HCl (*v*/*v*), washed three times with sterile water and then stained with 0.05% Trypan blue (*w*/*v*) or 5.0 μg/mL wheat germ agglutinin 488 (WGA488; Invitrogen, Carlsbad, CA, USA), according to the manufacturer’s instructions, respectively. AM fungal structures were observed with the appropriate microscopy. AM fungal spores and the stained fungal tissues were observed with a light microscopy (Nikon Y-TV55). Fluorescent signals in AM fungal spores and mycorrhizal roots were examined using a fluorescence microscopy (Nikon Y-TV55). The fungal spores were captured by a stereomicroscope (Nikon DS-R12). A Zeiss 780 laser scanning confocal microscope equipped with × 63 water immersion objective was used for the detection of arbuscules within roots. The excitation/emission of WGA488 were 488 nm/519 nm, respectively.

## 3. Results and Discussion

### 3.1. Carbon Sensing and Signaling

**Figure 1:**
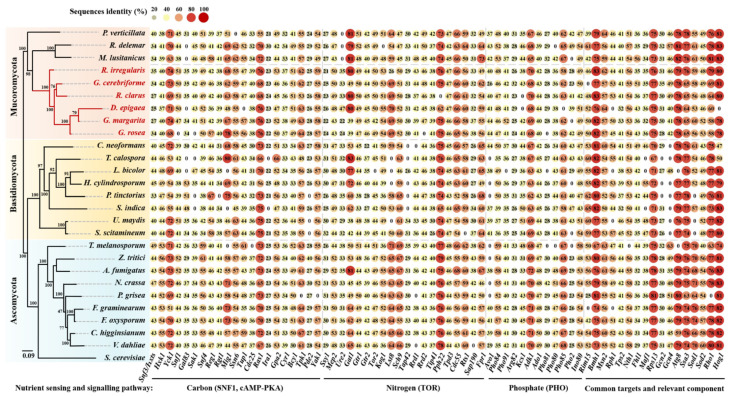
Heatmap of sequences identities. Amino acid sequences of the 70 components of nutrient sensing and signaling pathways in the 26 fungal genomes share identities with those in *S. cerevisiae*. The data (as a percentage, %) in dots represent the identities that were carried out when the sequences derived from yeast were used as the queries for genome BLAST searching. All hits producing identities greater than 20% and E-values below 10^−4^ were analyzed. The phylogenetic tree was reconstructed from the concatenated alignment of these genes using maximum-likelihood (ML) algorithms (RAxML) with the LG + I + G4 + F substitution model. The heatmap of identities was visualized by the TBtools [63]. Local BLAST results and detail information including accession numbers are shown in Supplemental Table S1.

**Figure 2:**
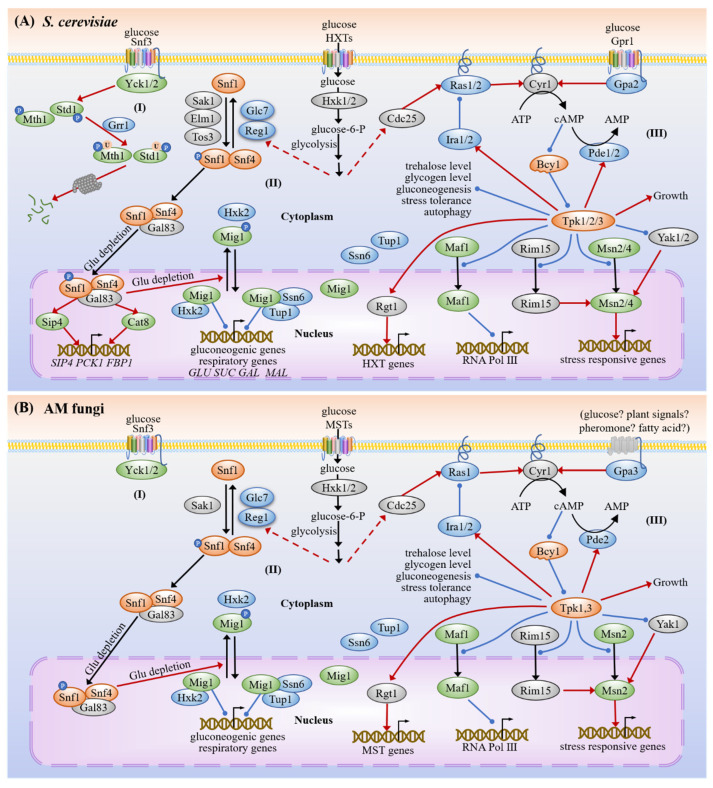
Carbon sensing and signaling pathways in yeast (**A**) and AM fungi (**B**). In the presence of glucose, Snf3/Rgt2 and Tpk3-dependent protein kinase trigger the induction of hexose transporter genes expression via Rgt1. The hexose transporters transport glucose into the fungal cell. Then, the Hxk1 and Hxk2 promote an increase in the intracellular glucose content. During glucose starvation, the activated Snf1 interacts with Snf4 and one of β-subunits Gal83/Sip1/Sip2 to form the SNF1 complex, which prevents the nuclear localization of both Hxk2 and Mig1. In the presence of glucose, Mig1 associates with its co-repressors Ssn6 and Tup1 to repress the expression of genes related to the availability of alternative carbon sources, such as sucrose, maltose and galactose, and regulates the transcription of the monosaccharide transporter genes (*MSTs*) in AM fungi. In the cAMP-PKA cascade, both Ras systems composed of Cdc25-Ras1-Ira1/2 sensing the intracellular glucose, and the Gα subunit Gpa2/3 sensing extracellular glucose, can activate the Cyr1 to generate cAMP with ATP as the substrate. Then, cAMP binds to Bcy1 and releases the Tpks, causing the activation of PKA. PKA suppresses cAMP accumulation via Ira1/2 and Pde2. On the other hand, PKA can directly phosphorylate the trehalase that is responsible for the rapid changes in trehalose levels and prevent the entrance of Maf1, Rim15 and Msn2 to the nucleus; thus, it regulates the expression of genes involved in Pol III activity, stress-response, tolerance and cell life span. In AM fungi, the extracellular glucose, plant signals, pheromone and fatty acid may activate cAMP-PKA via G protein α subunits Gpa3. AM fungi have two PKA catalytic subunits, Tpk1 and Tpk3, while the protein kinases Mth1, Std1, Grr1, Elm1 and Tos3 are not found in the genomes of AM fungi. The red lines with arrows indicate facilitation, whereas the blue lines with the dots indicate inhibition and the black arrows represent translocation or transformation.

The previous reports revealed that Snf3/Rgt2, cAMP-PKA and SNF1 pathways in yeast are mainly responsible for carbon sensing and signaling [27,28]. Here, we found that at least 22 orthologous proteins involved in these three pathways are conserved in AM fungi (Figure 1, Table 2) and their putative interactions are shown in Figure 2.

#### 3.1.1. Snf3/Hxts

The conserved domains of orthologous proteins of Snf3/Rgt2/Hxts in AM fungi (Table 2) indicate that they may have the abilities to bind the sugars, including glucose and xylose, but it cannot suggest that they act as the sugar transporters, as some of their homologs have lost the capacity to transport any substrate [64]. The binding of extracellular glucose with its sensor Snf3/Rgt2 can indirectly result in the exposure of Rgt1 via Yck1 and the phosphorylation of Rgt1 by PKA, or by a Tpk3-dependent protein kinase [65,66]. Both of these two processes driven by Snf3/Rgt2 can lead to the conversion of Rgt1 from a repressor into an activator that triggers the induction of *HXT* genes encoding hexose transporters [67].

More recent study has shown that *S. cerevisiae* can sense extracellular high xylose and alter the expression of *HXT2* via Snf3 [68]. In *R. irregularis*, xylose serves as an alternative carbon source and also functions as a signal to trigger the expression of the versatile monosaccharide transporter gene *MST2* in the extraradical mycelium [16], suggesting that sugars can act as signals to regulate the sugar uptake of AM fungi. When AM fungi are exposed to high extracellular sugar levels, it seems that it is the transceptor that induces the expression of sugar transporter genes through relevant signaling pathways, such as cAMP-PKA, SNF1 and TOR [67,69,70]. When the hexose transporters HXTs take up glucose into the fungal cell, the hexokinases Hxk1 and Hxk2 phosphorylate glucose to glucose-6-P in the first step of glycolysis, resulting in an increase of intracellular glucose levels [71].

#### 3.1.2. SNF1

The sucrose non-fermenting kinase Snf1, a member of the Snf1/AMP activated protein kinase (AMPK) family, regulates multiple processes in response to low glucose or alternative carbon sources limitation [27]. In the presence of glucose, Snf1 is inactivated by its C-terminal autoinhibition, while it is activated by Snf4 binding to its autoinhibitory domain and interacting with one of β-subunits Gal83/Sip1/Sip2 to form the heterotrimeric complex SNF1 under glucose limitation and high levels of ADP [72,73,74].

**Figure 3:**
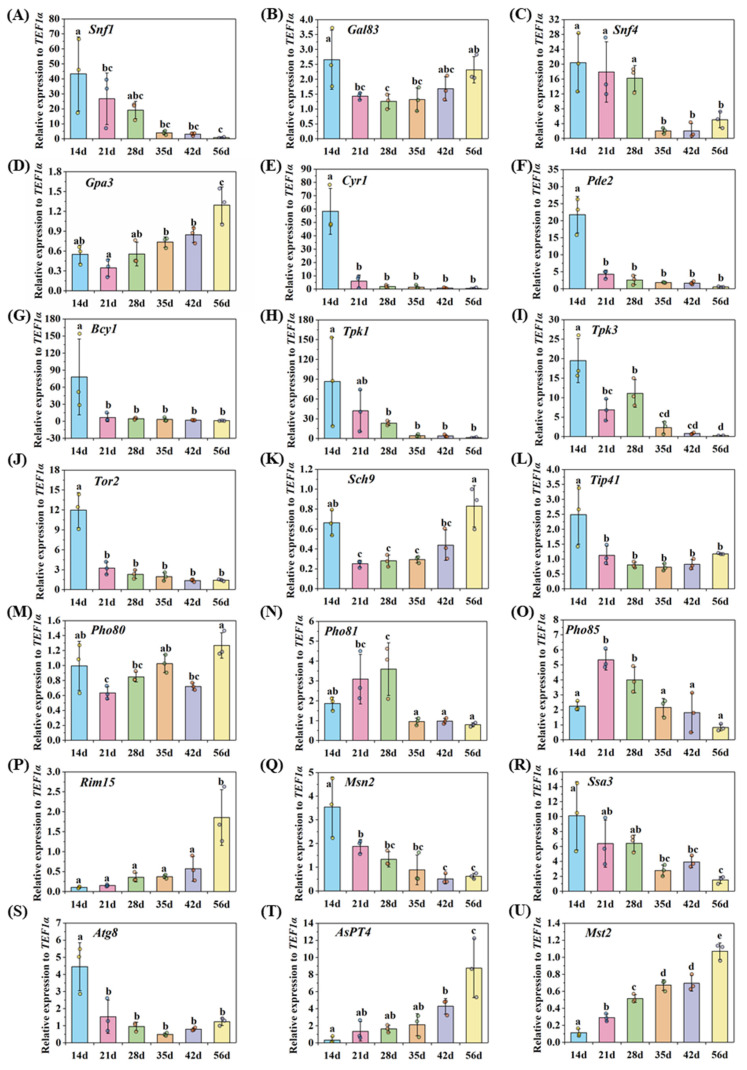
The expression patterns of the key AM fungal genes involved in the nutrient signaling pathways and symbiotic marker genes in the *A. sinicus* mycorrhizal roots 14, 21, 28, 35, 42 and 56 days (d) post inoculation with *R. irregularis*. (**A**–**U**) The transcript levels of target genes were estimated by real time qRT-PCR. (**A**–**C**) *Snf1*, *Gal83* and *Snf4* in the SNF1 pathway. (**D**–**I**) *Gpa3*, *Cyr1*, *Pde2*, *Bcy1*, *Tpk1* and *Tpk3* in the cAMP-PKA pathway. (**J**–**L**) *Tor2*, *Sch9* and *Tip41* in the TOR pathway. (**M**–**O**) *Pho80*, *Pho81* and *Pho85* in the PHO pathway. (**P**–**S**) The common targets *Rim15*, *Msn2*, *Ssa3* and *Atg8*. (**T**–**U**) The symbiotic marker genes *AsPT4* and *RiMst2*, whose expression patterns indicate the arbuscular mycorrhizal development. The *RiEF1α* (*translation elongation factor 1α* in *R. irregularis*) was used as the reference gene for normalization of AM fungal genes expression, while *AsActin* (*β-actin* in *A. sinicus*) was used as the reference gene for normalization of the *A. sinicus* gene *AsPT4* expression. Error bars represent the means of three biological replicates with ± SD values. Different letters indicate significant differences at *p* < 0.05, according to the Duncan’s test.

The RNA-seq data showed that the expression level of the *Snf4* of *G. rosea* in mycorrhizal roots was significantly down-regulated, compared with that in spores (Table 2). Further qRT-PCR results reveal that the expression levels of *Snf1* and *Snf4* of *R. irregularis* were highest at the early stage during symbiosis, suggesting these genes may play important roles in appressorium formation and penetration (Figure 3A,C), whereas the expression pattern of *Gal83* indicates that it may play roles in the early colonization and arbuscule development during symbiosis (Figure 3B).

The activation of Snf1 requires phosphorylation by Sak1 [75]. Glc7-Reg1 dephosphorylates Snf1, possibly leading to glucose-sensing via the Hxk2 [28,76]. Hxk2 binds to Mig1 (multicopy inhibitor of GAL gene expression) to prevent SNF1 from stimulating the translocation of Mig1 into cytoplasm at high glucose levels, whereas SNF1 prevents the nuclear localization of both Hxk2 and Mig1 by phosphorylation upon glucose exhaustion [77].

In the presence of glucose, Mig1 associates with its co-repressors Cyc8/Ssn6 and Tup1 to inhibit the expression of the glucose-repressed genes related to the availabilities of alternative carbon sources, such as sucrose (*SUC2*), maltose (*MAL*) and galactose (*GAL*) [78]. Snf1-Mig1 affects *HXTs*/*MSTs* transcription by repressing the expression of the regulators of Rgt1 [79]. The SNF1 regulates spore germination, hyphal growth, stress tolerance, colonization and sporulation in filamentous fungi (Supplemental Table S2), while its roles in AM fungi should be further studied.

#### 3.1.3. cAMP-PKA

The cAMP-dependent PKA consists of two regulatory subunits encoded by the *Bcy1* and two catalytic subunits encoded by the *Tpk1-3* in *S. cerevisiae* [80,81]. Most filamentous fungi possess two catalytic subunits and it is likely that only one subunit plays a predominant role [82,83,84]. AM fungi have two PKA catalytic subunits, Tpk1 and Tpk3, based on the genomic analysis. Both Ras systems composed of Cdc25-Ras1-Ira1/2 sensing the intracellular glucose, and the G protein α subunit Gpa2/3 sensing extracellular glucose, can activate the adenylate cyclase Cyr1 to generate cAMP with ATP as substrate [85,86,87,88,89]. cAMP binds to Bcy1 and then releases the Tpks, causing the activation of PKA signaling. PKA suppresses cAMP synthesis via Ira1/2 and promotes the cAMP hydrolysis via the phosphodiesterase Pde2, in order to down-regulate cAMP accumulation and maintain metabolic balance [90,91].

The RNA-seq data showed that the transcript levels of the *Pde2* of *R. irregularis* and the *Cyr1* of *G. rosea* were significantly up-regulated in mycorrhizal roots (Table 2), indicating an enhancement of both synthesis and degradation of cAMP and the balance of PKA activity. The highly induced expression levels of several PKA-related genes (*Cyr1*, *Pde2*, *Bcy1*, *Tpk1* and *Tpk3*) of *R. irregularis* were detected at the early stage during symbiosis by qRT-PCR (Figure 3E–I), suggesting that these genes may play important roles in the appressorium formation and penetration. The penetration process has been shown to be mediated by degradation of triacylglycerol and glycogen [29,92]. On the basis of the biotrophic interactions of both symbiotic fungi and pathogenic fungi with the host plants, they go through many similar processes during their growth and development, including signaling exchange and host cell penetration [93,94]. Therefore, the functions of some genes in these biotrophic fungi may be similar.

The dominant active *GPA3* of *Umbilicaria muhlenbergii*, a fungus forming symbiosis with *Trebouxia jamesii*, led to the enhanced pseudohyphal growth and the death of *T. jamesii* [95]. This indicates that the down-regulated expression of the putative *Gpa3* of *R. irregularis* and *G. rosea* in mycorrhizal roots showed by RNA-seq data (Table 2) was beneficial to accommodate the symbiotic interaction. In addition, Gpa3 may sense external signals (such as pheromone, glucose, or lipid) from the hosts or the environments [87,88,89]. According to the expression pattern of *Gpa3* of *R. irregularis* in mycorrhizal roots detected by qRT-PCR (Figure 3D), we proposed that *Gpa3* may contribute to the signal induction, accommodation and symbiosis development.

cAMP-PKA directly or indirectly regulates the expressions of nearly 500 genes in cells [96]. PKA negatively regulates Yak1 kinase, which phosphorylates transcription factor Msn2, but does not influence the nucleus localization of Msn2 [97]. PKA prevents the entrance of Maf1, Rim15 and Msn2 into the nucleus to negatively regulate the expression of genes involved in Pol III activity, stress responses and fungal life span [98,99]. cAMP-PKA affects a variety of physiological processes, including autophagy, lipid metabolism, spore germination, mycelium growth, stress tolerance, appressorium formation, colonization and sporulation in filamentous fungi (Supplemental Table S2), but its detailed roles in AM fungi remain unclear.

### 3.2. Nitrogen Sensing and Signaling

The constitutive expression of *AMT2* induced the expression of *AMT1* of *G. intraradices* under nitrogen limitation and this may be similar to nitrogen catabolite repression (NCR) in *S. cerevisiae* [10]. NCR is regulated by Gln3 and Ure2 involved in the TOR pathway [27,100]. TOR in *S. cerevisiae* has two Tor kinases, namely, Tor1 and Tor2, while most fungi contain only one of them [101]. Either Tor1 or Tor2 can associate with Kog1, Lst8 and Tco89 to form TOR complex 1 (TORC1), while only Tor2 can also associate with Avo1-3, Bit61 and Lst8 to form TOR complex 2 (TORC2) [102,103]. Rapamycin can bind to its receptor FKBP12 encoded by *FPR1* to form a complex, which further binds to Tor1/2, thereby inhibiting the rapamycin-sensitive TORC1 [103,104]. TORC1 and TORC2 are conserved among distinct AM fungi and it seems that only one Tor kinase, Tor2, is present (Figure 1, Table 2). It was reported that the *GmTOR2* of *Glomus mosseae* (currently *F. mosseae*) was expressed in the sporocarps, mycorrhizal roots and extraradical hyphae [105].

**Figure 4:**
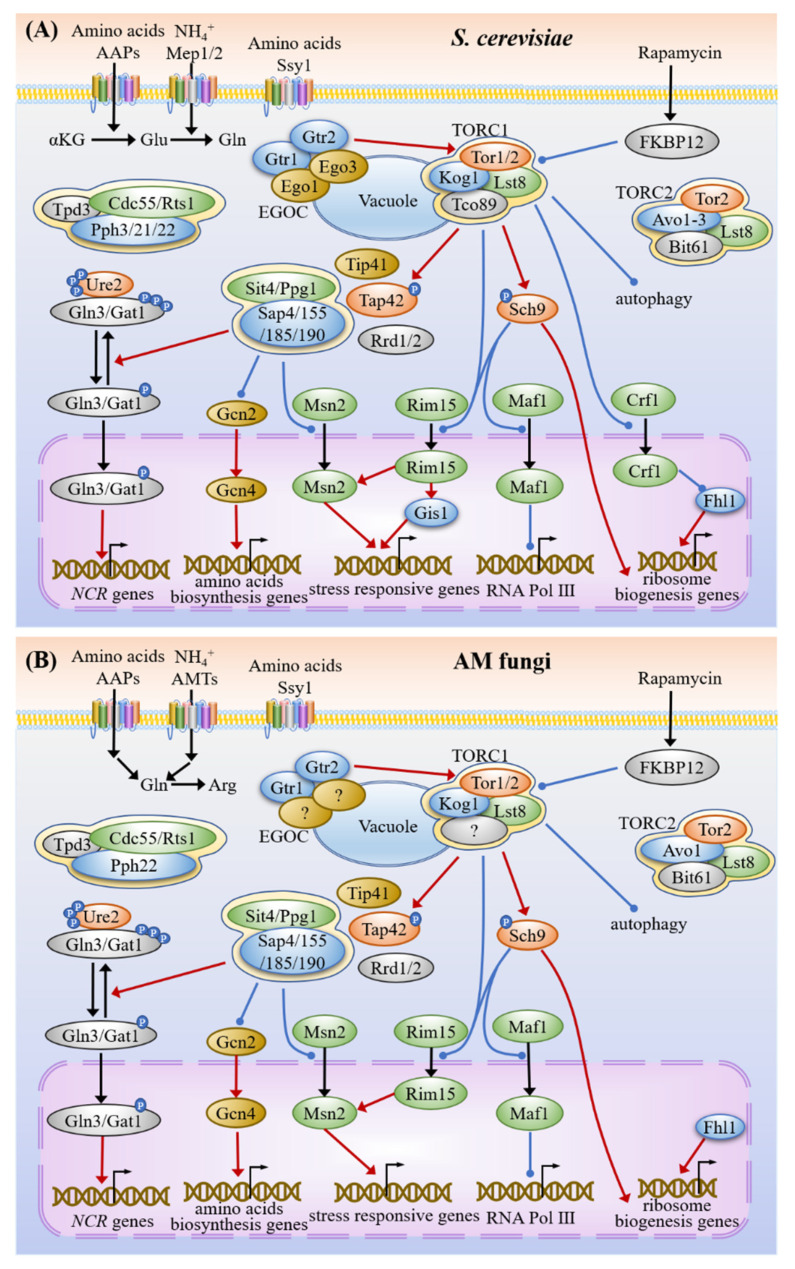
TOR signaling pathways in yeast (**A**) and AM fungi (**B**). The amino acids are sensed by Ssy1 and transported by the amino acid permeases (AAPs), while the ammonium is sensed and transported by AMTs/Meps. The increase in amino acids levels also activates TORC1 via the EGO complex, which is composed of Gtr1, Gtr2 and other unknown proteins. TORC1 is localized to the vacuolar membrane, consisting of Tor2, Kog1 and Lst8. The activated TORC1 phosphorylates the downstream targets Sch9 and Tap42. Tap42 associates with phosphatase PP2A (Pph22, Tpd3 and Cdc55/Rts1) and PP2A-related protein phosphatases (Sit4/Ppg1 and Sap190) complexes, along with the regulatory protein Rrd1/2. TORC1 enhances the hyperphosphorylation and association of Gln3/Gat1 and Ure2, preventing the nuclear localization of Gln3/Gat1 via the Tap42-PP2A complex, resulting in the suppression of genes related to nitrogen catabolite repression (NCR) and use of less preferred nitrogen sources. Tip41 interacts with Tap42 to negatively regulate the TOR pathway. Rapamycin binds to its receptor FKBP12 encoded by *FPR1* to form a complex, which can bind Tor2, thereby inhibiting TORC1. TORC1 promotes Sch9 activity that can phosphorylate Maf1 to activate RNA polymerase III-transcribed genes. Tap42 effector positively regulated by TORC1 prevents the translation of Gcn4, a transcriptional activator needed for amino acid biosynthesis. Tor2 also can bind to Avo1, Sit4 and Bit61 to form the TORC2 complex. The proteins Tco89, Ego1 and Ego3 are not found in the genomes of AM fungi. The red lines with the arrows indicate facilitation, whereas the blue lines with the dots indicate inhibition and the black arrows represent translocation or transformation.

We found that at least 21 orthologous proteins involved in the TOR pathway are conserved in AM fungi (Figure 1, Table 2). By referring to the studies in yeasts, their putative interactions and functions in AM fungal cells were shown in Figure 4. The amino acids are sensed by the senor Ssy1 and transported by the amino acid permeases (AAPs), while the ammonium is transported by transceptors Meps or transporters AMTs [10,106,107,108,109]. The increase of amino acids can activate TORC1 via the EGO complex [110]. The activated TORC1 located into the vacuolar membrane can phosphorylate the main downstream targets Sch9 and Tap42 [102]. Tip41 interacts with Tap42 to negatively regulate the TOR pathway [111].

The transcripts of AM fungal *Tor2*, *Sch9* and *Tip41* were down-regulated in mycorrhizal roots compared with those in spores shown by RNA-seq (Table 2), and the down-regulated expression levels of *Tor2* and *Tip41* of *R. irregularis* with prolongation of the symbiotic stage were detected by qRT-PCR (Figure 3J,L). These results suggest that TOR signaling may play important roles in the appressorium formation and fungal development, which were known to be under the regulation of lipid droplet biogenesis required for fungal triacylglycerol accumulation [112]. Besides, Sch9 could regulate the spore germination, hyphal growth and branching, and secondary metabolism in fungi [113]. The distinct expression of *Sch9* in *R. irregularis* during different stages indicates that *Sch9* may play important roles in the early colonization and arbuscule development (Figure 3K).

As a component of downstream of TORC1, Tap42 associates with phosphatase PP2A (Pph3/21/22, Tpd3 and Cdc55/Rts1) and PP2A-related protein phosphatases (Sit4/Ppg1 and Sap4/155/185/190) complexes along with the regulatory proteins Rrd1/2 [114,115]. TORC1 enhances the hyperphosphorylation and association of the Gln3/Gat1 and Ure2 to prevent the nuclear localization of Gln3/Gat1 via the Tap42-PP2A complex, leading to the suppression of NCR genes related to the utilization of alternative nitrogen sources [100]. TORC1 and Sch9 can phosphorylate Maf1 to activate RNA polymerase III-transcribed genes [116]. Tap42 effector branch prevents the phosphorylation of eIF2α by Gcn2, thereby inhibiting the translation of Gcn4, a transcriptional activator needed for amino acid biosynthesis in response to amino acid starvation [117].

The TOR pathway regulates a series of processes, including stress responses, secondary metabolism, appressorium formation, differentiation, virulence and sporulation in multiple fungi species (Supplemental Table S2), but its roles in AM fungi remain elusive.

### 3.3. Phosphate Sensing and Signaling

**Figure 5:**
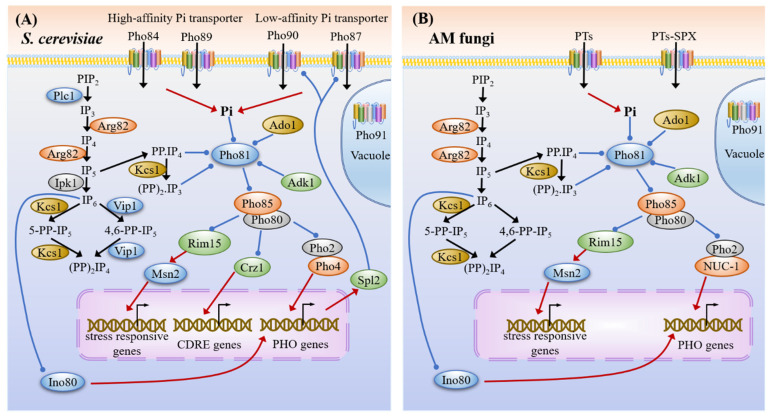
PHO signaling pathways in yeast (**A**) and AM fungi (**B**). The phosphate transporters take up the external phosphate into AM fungi. The inositol-polyphosphate kinase Arg82 and inositol hexakisphosphate (IP_6_) kinase Kcs1 involved in the inositol phosphate metabolism can mediate the increase of IP_7_, which is in response to low phosphate and functions upstream of Pho81, a mediator of intracellular phosphate sensing in the PHO pathway. The adenylate kinase Adk1 and adenosine kinase Ado1 affect IP_7_ synthesis and function upstream of Pho81 to negatively regulate the expression of *PHO* genes. The interaction between IP_7_ and Pho81 induces the formation of the Pho81 and Pho85-Pho80 tri-complex, resulting in the nuclear localization of the transcriptional activator NUC-1 (Pho4 homolog) with cofactor Pho2. Subsequently, the downstream of genes involved in the PHO pathway is transcriptionally activated under phosphate limitation. Under high phosphate conditions, the SPX domain-containing phosphate transporters PTs in AM fungi (e.g., RiPT7 in *R. irregularis* and GigmPT6/7 in *G. margarita*, homologs of Pho87/90 in yeast) are responsible for the phosphate transport and homeostasis. The phosphorylation of NUC-1 by the Pho85-Pho80 complex and its export by Msn5 out of the nucleus cause the repression of the PHO pathway. Ino80, whose ATPase activity is inhibited by IP_6_, is required for the regulation of chromatin remodeling and transcription, including the expression of some PHO genes. The homologs of SPX domain-containing proteins PTs-SPX can regulate phosphate homeostasis at arbuscular mycorrhizas, while the vacuolar transporter chaperones (VTCs) and polyphosphatases Ppn1 and Ppx1 can regulate vacuolar polyphosphate metabolism. The proteins Plc1, Ipk1, Spl2 and Crz1 are not found in the genomes of AM fungi. The red lines with the arrows indicate facilitation, whereas the blue lines with the dots indicate inhibition and the black arrows represent translocation or transformation.

The PHO pathway is responsible for phosphate sensing and signaling in fungal cells [27,28]. The genome of *G. margarita* contains a set of core components involved in the PHO pathway [38]. We found that at least 12 core components involved in this PHO pathway are conserved in AM fungi (Figure 1, Table 2), and their putative interactions and functions are shown in Figure 5.

The phosphate transporters PTs take up the external phosphate into the fungi. Under high phosphate conditions, the SPX domain-containing phosphate transporters PTs in AM fungi (e.g., RiPT7 in *R. irregularis* and GigmPT6/7 in *G. margarita*, orthologs of Pho87/90 in yeast) are responsible for phosphate transport [38]. While the homologs of SPX domain-containing proteins can regulate phosphate homeostasis, some vacuolar transporter chaperones (VTCs) and polyphosphatases Ppn1/Ppx1 can regulate vacuolar polyphosphate metabolism [118,119,120].

In fungal cells, the inositol-polyphosphate kinase Arg82 and inositol hexakisphosphate (IP_6_) kinase Kcs1 that are involved in the inositol phosphate metabolism mediate the increase of IP_7_. IP_7_ is responsible for low phosphate and functions upstream of Pho81, a mediator of intracellular phosphate sensing [121,122,123]. The adenylate kinase Adk1 and adenosine kinase Ado1 can affect IP_7_ synthesis and function upstream of PHO81 to negatively regulate *PHO* genes expression [124,125]. The interaction between IP_7_ and Pho81 induces the formation of the Pho81 and Pho85-Pho80 tri-complex, resulting in the nuclear localization of the transcriptional activator NUC-1 (Pho4 homolog in yeast) with cofactor Pho2 [126,127].

Subsequently, the downstream genes involved in the PHO pathway are transcriptionally activated under phosphate limitation [128]. The phosphorylation of NUC-1 by the Pho85-Pho80 complex [129] and its export out of the nucleus by Msn5 [130] cause the repression of PHO pathway. Ino80, whose ATPase activity is inhibited by IP_6_, is required for the regulation of chromatin remodeling and transcription, including the expression of some *PHO* genes [131,132].

The RNA-seq analysis showed that the expression levels of phosphate transporters encoding genes (*Pho84* and *Pho91*) of *R. irregularis* and *G. rosea*, and phosphate responsive genes of *R. irregularis* (*Kcs1*, *Ado1*, *Pho81* and *Pho2*) and *G. rosea (Adk1*, *Pho2*, *Pho4* and *Pho85*) in mycorrhizal roots, were significantly up-regulated when compared with those in spores (Table 2). This indicates that the phosphate transport and metabolism of AM fungi were enhanced in order to increase the phosphate supply provided to plants during symbiosis.

Furthermore, the qRT-PCR results indicate that the PHO regulators (*Pho80*, *Pho81* and *Pho85*) of *R. irregularis* in mycorrhizal roots may play pivotal roles in symbiotic phosphate uptake and homeostasis during AM symbiosis (Figure 3M–O). The PHO pathway regulates a series of processes, including fungal growth, phosphate uptake and homeostasis, stress tolerance and sporulation in numerous fungi species (Supplemental Table S2) and its roles in AM fungi need to be extensively conducted.

### 3.4. Common Targets and Crosstalk

**Figure 6:**
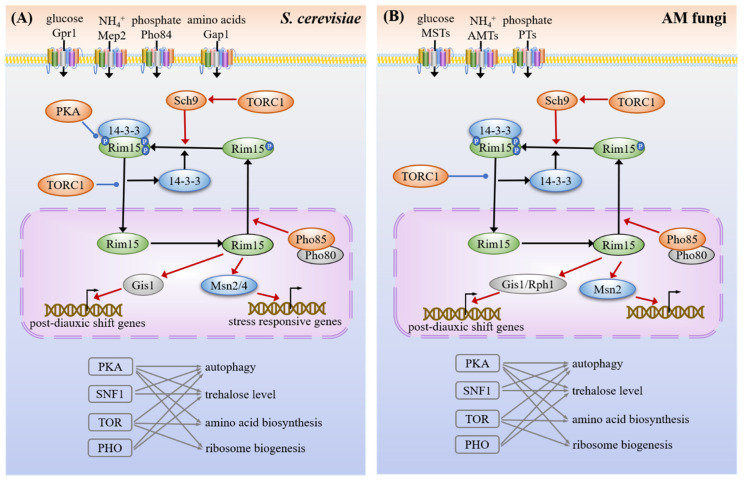
Common targets and crosstalk among nutrient signaling pathways in yeast (**A**) and AM fungi (**B**). The nutrient transporters acquire various nutrients from the environments and some transporters, which also act as the transceptors (transporters and receptors), transport nutrients as well as activate nutrient signaling pathways, including cAMP-PKA, TOR and PHO. All the pathways of the PKA, TORC1, Sch9 and PHO converge on the serine/threonine protein kinase Rim15. Rim15 interacts with the 14-3-3 protein to regulate Msn2/4 and Gis1 transcription factors (TFs), thereby activating the expression of the stress-responsive element (STRE) genes and post-diauxic shift (PDS) genes, respectively. The crosstalks among these pathways share a large number of common downstream targets. Msn2 and Gis1 TFs activate the transcription of the genes encoding heat shock proteins (e.g., *Hsp12*, *Hsp26*, *Ssa3*) upon nutrient limitation. The PKA, SNF1, TOR and PHO pathways control the expression of genes involved in autophagy (e.g., *Atg8*, *Atg11*, *Atg13*). The PKA, SNF1 and PHO pathways also control the expression of genes involved in the trehalose metabolism (e.g., *Tps1*, *Tps2*, *Nth1*), while the PKA and TOR pathways control the expression of genes involved in amino acid biosynthesis (e.g., *Gcn2*, *Gcn4*) and ribosome biogenesis (e.g., *Fhl1*, *Maf1*, *Rps*). The yeasts have both Msn2 and Msn4, while it seems that only Msn2 is present in the genomes of AM fungi. The red lines with the arrows indicate facilitation, whereas the blue lines with the dots indicate inhibition and the black arrows represent translocation.

The nutrient signaling pathways (cAMP-PKA, SNF1, TOR and PHO) can respond to multiple available nutrients, including but not limited to carbon, nitrogen and phosphate [133,134,135]. By referring to the studies on yeasts, we found that at least 17 common components involved in these four nutrients signaling pathways are conserved in AM fungi (Figure 1, Table 2), and their putative interactions and functions are shown in Figure 6.

The nutrient transporters can take up nutrients from the external environments into the fungal cells and the transceptors (e.g., homologs of Pho84 and Mep2) and sensors (e.g., homologs of Snf3 and Ssy1) can activate nutrient signaling pathways, including cAMP-PKA, TOR and PHO. All the four pathways of the PKA, TORC1, Sch9 and PHO converge on the serine/threonine protein kinase Rim15. Rim15 interacts with the 14-3-3 protein to regulate Msn2 and Gis1 transcription factors (TFs), thereby activating the expression of the STRE genes and post-diauxic shift (PDS) genes, respectively [28,135,136]. Msn2 and Gis1 can activate the transcription of the genes encoding heat shock proteins (e.g., *Hsp12*, *Hsp26*, *Ssa3*) upon nutrient limitation [137]. The PKA, SNF1, TOR and PHO pathways can regulate the expression of genes involved in autophagy (e.g., *Atg8*, *Atg11*, *Atg13*) and trehalose metabolism (e.g., *Tps1*, *Tps2*, *Nth1*), while the PKA and TOR pathways control the expression of genes involved in amino acid biosynthesis (e.g., *Gcn2*, *Gcn4*) and ribosome biogenesis (e.g., *Fhl1*, *Maf1*, *Rps*) [138,139,140,141,142,143,144,145,146,147,148].

The RNA-seq results show that the expression levels of *Rim15* of *R. irregularis* and *Msn2*, *Ssa3*, *Atg8* of *G. rosea* in mycorrhizal roots were significantly up-regulated, when compared with those in spores (Table 2). The expression pattern of *RiRim15* detected by qRT-PCR was accompanied by induction of the symbiotic marker genes *AsPT4* and *RiMst2* [16,61], which were strongly activated in arbusculated cells (Figure 3P,T–U). This implied that the AM fungal protein kinase Rim15 may play a crucial role in arbuscule development or arbuscule life span. The expression levels of *Msn2*, *Ssa3* and *Atg8* were highly expressed at the early stage of AM symbiosis, suggesting that these three genes may play important roles in the appressorium formation and fungal penetration (Figure 3Q–S). In conclusion, the common signal components mentioned above may be involved in the fungal penetration or arbuscule life span during AM symbiosis.

These pathways (cAMP-PKA, SNF1, TOR and PHO) have complex crosstalks in fungi. TORC1 and PKA negatively regulate the Snf1 [77,149,150,151]. On the contrary, the active Snf1 inhibits Cyr1 to negatively regulate PKA activity [152] and phosphorylates Kog1 to trigger disassembly of TORC1 under nutrient limitation [153]. TORC1 can function as an upstream activator of PKA via the inhibition of Bcy1 in a Sch9-dependent manner [154]. TORC1 and Snf1 function in parallel upstream of Sch9 to affect the growth and replicative life span [155]. Snf1 and Tpk3, or a Tpk3-dependent protein kinase, can affect the expression of *Hxk2* by the repression of Rgt1 activity [65]. On the other hand, phosphate transceptor Pho84 can activate the PKA signaling cascade in yeast [156,157]. Snf1 can activate Msn2/4 to regulate the expression of the vacuolar iron importer gene *Ccc1* in iron resistance [158].

In addition, other interactions beyond the above four nutrients signaling pathways have emerged in fungal cells. For example, TORC1 functions as both the regulator and the target of Rho1, a core sensor component of the CWI (cell wall integrity) pathway in fungi [159]; Sch9 acts as the mediator of TOR and HOG (high osmolarity glycerol) pathway [113]; Pde2 functions upstream of the MAP (mitogen-activated protein) kinase pathway to regulate CWI and can also mediate the crosstalk between the cAMP-PKA and HOG pathway [160]; Sod2 functions downstream of Sch9 to extend fungal longevity [161].

Therefore, these pathways share a lot of common components and downstream targets, including the genes involved in autophagy, trehalose metabolism and amino acids and ribosome biogenesis [27,28,146,162]. They can regulate various physiological processes ranging from fungal proliferation, stress resistance, longevity to sporulation. These findings suggest that there exist the complexity and diversity of crosstalks among these nutrients signaling pathways in AM fungi.

Several targets of these signaling pathways have been confirmed in AM fungi. Trehalose-6-phosphate synthase subunit Tps2 and neutral trehalase Nth1 responded to the heat shock in both *R. irregularis* and *F. mosseae* [163]. The gene encoding the 14-3-3 protein was up-regulated at the appressorium stage in *F. mosseae* [164]. In *Rhizoglomus irregulare*, the transcript levels of *14-3-3* and *HSP70* in roots at 4 days post inoculation (dpi) were higher than those at 16 dpi [165]. 14-3-3 proteins from *F. mosseae* and *R. irregularis* were shown to be involved in arbuscule formation and responses to abiotic stresses [62]. The expression of the probable autophagy protein encoding gene *Atg8* of *G. intraradices* was detected in the cortical cells containing arbuscules [166]. The expression levels of Cu/Zn superoxide dismutase gene *SOD* were up-regulated in *M. truncatula* mycorrhizal roots inoculated with *G. margarita* or *G. intraradices*, compared with those in spores [167,168].

As the macro-nutrient elements, carbon, nitrogen and phosphorus can interact with each other to regulate the growth and development of AM fungi during symbiosis. Nitrate stimulated the polyphosphate accumulation and the germ tube growth of *G. margarita* spores [169]. Carbon supply stimulated the fungal uptake and the transport of both phosphate and nitrogen in the AM symbiosis [170,171]. The supply of carbon could regulate the expression of the fungal ammonium and phosphate transporter encoding genes [10,14]. These results indicated that there exist crosstalks among different nutrient signaling in AM fungi.

The *Medicago truncatula mtpt4* mutants defective in plant phosphate acquisition from the AM fungus displayed premature arbuscule degeneration (PAD) within roots [172]. Low nitrogen treatment can suppress this PAD, while the ammonium transporter gene *ATM2:3* was required for the suppression of this PAD in *mtpt4* mutants [173]. Phosphate availability significantly affected mycorrhizal development and high phosphate treatments led to an inhibition of mycorrhizal development at the early stage [174,175]. On the other hand, high phosphate addition notably and temporarily restrained the development of new arbuscules, instead of the maintenance of arbuscules, and contributed to more vesicles with lipid droplets accumulation that decreased in a few days [176].

These findings raise open questions on the mechanisms of nutrients interactions and homeostasis in the coordination of AM fungal growth, arbuscule life span and sporulation during symbiosis. It would be interesting to reveal the roles of the common targets and crosstalk of these pathways (cAMP-PKA, SNF1, TOR and PHO) in AM fungi and AM symbiosis.

### 3.5. Nutrient Homeostasis in the Life Cycle of AM Fungi

**Figure 7:**
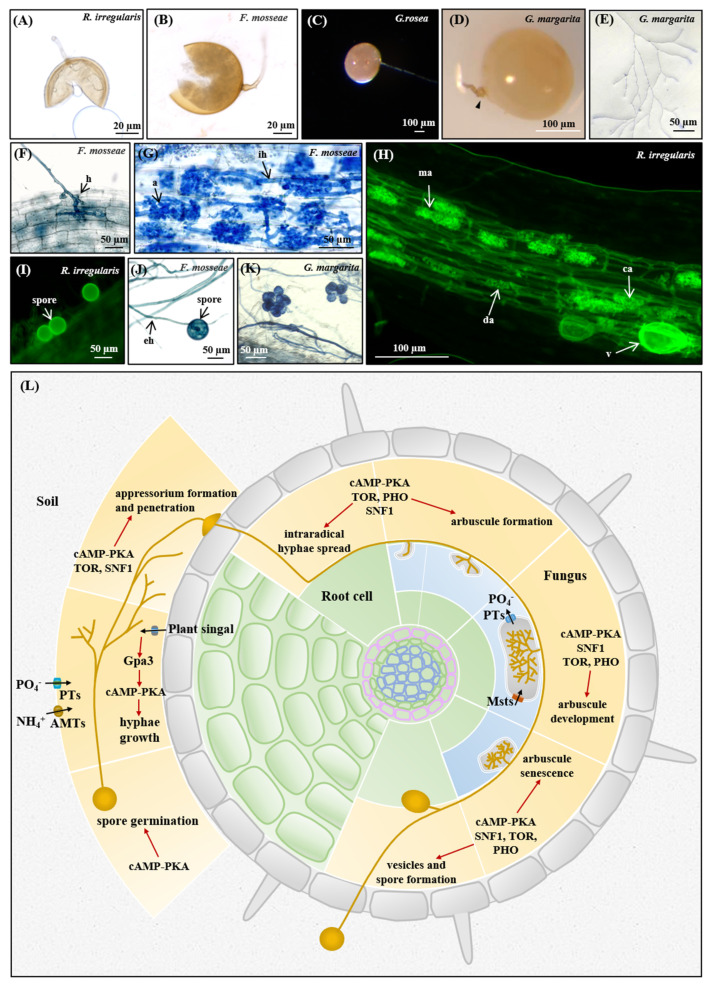
Potential functions of nutrient sensing and signaling pathways in the life cycle of AM fungi. The spores of *R. irregularis* (**A**), *F. mosseae* (**B**), *G. rosea* (**C**) and *G. margarita* (**D**). The branching hyphae of *G. margarita* (**E**). The hyphopodium of *F. mosseae* (**F**). The arbuscules of *F. mosseae* (**G**) and *R. irregularis* (**H**). The offspring spores of *R. irregularis* (**I**) and *F. mosseae* (**J**). The auxiliary cells of *G. margarita* (**K**). h, hyphopodium; a, arbuscule; ma, mature arbuscule; ca, collapsed arbuscule; de, dead arbuscule; ih, intraradical hyphae; eh, extraradical hyphae; v, vesicle. The pattern diagram (**L**) shows that the cAMP-PKA, SNF1, TOR and PHO pathways may regulate the spore germination, hyphal growth, appressorium formation and penetration, nutrients exchange and homeostasis, formation of vesicles (in Glomeromycotina fungi, except for the Gigasporales) or auxiliary cells (in Gigasporaceae fungi) and sporulation of AM fungi, as well as the development, senescence and life span of arbuscules. The black arrows represent translocation of transformation, while the red arrows represent positive regulation.

AM fungi are obligate biotrophs depending on the host plants for lipids and sugars to complete their life cycle [3,177]. This is due to their loss of the genes encoding type I fatty acid synthase required for long-chain fatty acid synthesis and their loss of the genes encoding glycoside hydrolases and invertase, that produces glucose [35,178]. With the help of host roots, they go through different stages, including spore germination, hyphae branching, appressorium formation and penetration, arbuscule formation and development and sporulation (Figure 7).

#### 3.5.1. Asymbiotic and Presymbiotic Stages

The life cycle of AM fungi begins with spore germination and its propagule growth during the asymbiotic stage (Figure 7A–D). In the germ tubes, lipid bodies were more abundant near the fungal spore [179]. The degradation, or conversion, of fatty acids from triacylglycerols to phospholipids was observed during germination and germ tube growth [180]. The conversion of lipid to trehalose during spore germination [181] and conversion of carbohydrates from hexose to trehalose and glycogen also exists [182]. The cAMP-PKA and SNF1 signaling pathways, which are required for germination and hyphal growth [183,184,185], can control the usage of carbon sources and lipid metabolism [186,187]. PKA can directly phosphorylate and activate trehalase, that is responsible for hydrolyzing trehalose into two glucose molecules [188].

During the presymbiotic period, the receptor-like kinases (e.g., LysM-RLKs, SYMRK/DMI2) in plant roots can perceive AM fungal signals (e.g., lipochito-oligosaccharides (LCOs), short-chain chitin oligosaccharides (COs)) to trigger common symbiotic signaling pathways that transmit fungal signals from the plasma membrane to the nucleus, leading to AM formation [22,189,190,191,192,193,194]. AM fungi sense strigolactones secreted by roots to stimulate the hyphal branching [195,196]. This may be similar to that in some plant pathogenic fungi—AM fungi may sense the plant signals via Gpa3 protein and G-protein-coupled receptors (GPCRs) to activate cAMP-PKA signaling [87,88,89,197,198]. It would be interesting and significant to reveal the roles of cAMP-PKA at the presymbiotic stage of AM fungi.

#### 3.5.2. Appressorium Formation and Penetration

AM fungi form appressorium on the surface of the plant roots for penetration into the epidermal cells. This process may be similar to some plant fungal pathogens [194]. Subsequently, the appressorium penetrates the epidermal cells to grow the hyphal tips; this penetrated structure is called hyphopodium (Figure 7F). This penetration requires the physical pressure (up to 8 Mpa) derived from the appressorial turgor, resulting from the accumulation of glycerol generated by the degradation of triacylglycerol and glycogen [92,199].

PKA regulates the degradation of triacylglycerol and glycogen to facilitate turgor generation and SNF1 regulates the lipid mobilization required for appressorium formation [29,92,185,187,200]. However, TOR signaling serves as an inhibitor, while rapamycin can induce the appressorium formation in the PKA mutants [201]. The appressorium formation also requires autophagic fungal cell death [202]. It is necessary to address the regulatory mechanism of the cAMP-PKA, TOR, SNF1 and their crosstalk on the autophagy-mediated lipid utilization and appressorium formation.

#### 3.5.3. Arbuscule Formation and Development

The intraradical hyphae run across the outer/inner cortex and develop the highly branched tree-like structures called arbuscules in the cortical cells (Figure 7G,H). The arbuscules with short life spans are considered to be the main sites of nutrient and signal exchanges between the symbiotic partners [203].

- Carbon-phosphate trade in arbuscules

Carbon delivered from plants to AM fungi are essential for the “carbon-phosphate trade” in mutualistic symbiosis [170,204]. The host plants release sugars into the peri-arbuscular space through the sugar transporters [205,206]. AM fungi take up sugars in the form of hexoses through the monosaccharide transporters (MSTs) with different substrate specificity [207]. In addition to sugars, the host plants also delivery lipids as a major organic carbon to AM fungi. The host plant activates *de novo* lipid biosynthesis, then releases them into the symbiotic interface space [2,208]. Both sugar and lipid can act as signals and carbon sources to promote the arbuscule formation and development, but there remains a lack of knowledge about the regulatory mechanism. It would be interesting to reveal the roles of cAMP-PKA and SNF1 in arbuscule formation during symbiosis.

AM fungi absorb external phosphate and then transfer it as polyphosphate into the intraradical hyphae and arbuscules via a motile tubular vacuole system and the aquaporin AQP3 [17,18]. The fungal polyphosphatases Ppn1 and Ppx1 can hydrolyze polyP into free Pi, which is exported to arbuscule cytoplasm from the intraradical hyphae by the unknown fungal vacuolar Pi efflux transporter, leading to arbuscules at a high Pi status [9,209,210]. The process of Pi efflux, from the arbuscules to the periarbuscular space (PAS), or apoplast, through a specialized efflux system, may exist in the intracellular and plasma membranes of arbuscules [9]. It has been proposed that the SPX (SYG1/Pho81/XPR1) domain-containing proteins and proton-coupled symporters may participate in Pi efflux process to regulate Pi homeostasis at symbiotic interface [9,211,212,213].

Therefore, Pi is unloaded through either of the following four hypothetical pathways: (1) Pi is released from the vacuoles to the cytosol via exporter PHO91 and loaded to the Golgi/trans-Golgi network by PHO1-type Pi transporter for unloading to the PAS or apoplast; (2) the cytosol Pi is directly unloaded by PHO1-type Pi transporter localized on the plasma membrane of hyphae; (3) PolyP is directly exported via the VTC1/2/4 complex, sorted to the fungal plasma membrane to the PAS or apoplast, then hydrolyzed by plant acid phosphatase [9,214]; (4) Pi is exported from the fungus through proton-coupled Pi transporters [212]. Finally, Pi released to the PAS is acquired by the arbuscular mycorrhiza-inducible plant PHT1 family Pi transporters localized in the peri-arbuscular membrane (PAM) [172,215,216]. However, the defined mechanisms by which AM fungi handle Pi homeostasis in arbuscules are poorly understood. 

Pi also functions as a signal molecule to regulate multiple response characteristics in AM symbionts [14,217]. The knockdown of phosphate transceptor gene *GigmPT* by the host-induced gene silencing (HIGS) strategy leads to a defect in arbuscule development with more collapsed arbuscules containing septa and further investigation indicates that Pi sensor GigmPT orchestrates AM development through the PKA and PHO signaling pathways [14]. This finding reveals that phosphate availability can regulate arbuscule development through nutrient sensing and signaling pathways in AM fungal symbionts. It would be of significance to reveal the precise roles of core components in the PKA and PHO pathways in AM fungi.

- Life span of arbuscule

The arbuscules have short life spans of 4–15 days before they collapse [24,176]. New arbuscules are constantly formed in order to maintain the substantial nutrient trade to complete the obligate biotrophic life cycle of AM fungi. Amount of membrane phospholipids is generated per arbuscule and these phospholipids consist of two phosphates and two fatty acids [218]. The lipid globules are seen in the senescent arbuscule trunk and branches with a chain of central vacuoles, and collapsed arbuscules are associated with lipid-rich intercellular hyphae [219]. The older intercellular hyphae, senescent arbuscules and offspring spores contain an amount of lipid droplets that appears to be derived from the preformed lipids, which may contribute to resource utilization and carbon-phosphate exchange [219,220].

More recent studies have shown that an arbuscule contains abundant membrane tubules, that are analogous structures formed by the invasive hyphae of maize pathogen *U. maydis*, whereas phosphoinositides accumulate in the distinct regions of the periarbuscular membrane [94,221]. This is similar to the biotrophic interfacial membrane complex in rice infected with *M. orzyae* [218]. Small anionic lipids with their specific subcellular allocation during arbuscule development are considered to influence the membrane trafficking and signaling, indicating these anionic lipids may regulate arbuscule development [24,218]. Therefore, it is proposed that the sustained development of arbuscule requires phosphate and lipids which may be involved in symbiotic nutrient exchange.

The TOR, PKA and PHO pathways can regulate phosphatide phosphatase activity of Pah1, which can promote the conversion from phosphatidic acid to diacylglycerol that acts as the precursor required for the synthesis of triacylglycerols and lipid droplets [112,222,223]. Moreover, Msn2 can activate the expression of the genes involved in fatty acid metabolism [224].

Low pH significantly reduces arbuscule formation and phosphate acquisition, resulting in more degenerating arbuscules with neutral lipid accumulation [225]. The cytosolic pH functions as a cellular signal to trigger the Ras/PKA and TORC1 in response to glucose levels [226]. The PKA, TORC1 and Sch9 pathways regulate the assembly of a highly conserved vacuolar proton pump (V-ATPase), a sensor for cytosolic pH, to mediate the acidification of multiple organelles, thereby regulating pH homeostasis, autophagy and fungal longevity [227,228,229].

In addition, cAMP-PKA, Sch9, TOR and Pho85 negatively regulate the fungal autophagy, whereas Snf1 has a positive effect on it, which is known to be essential for cellular longevity and aging [138,139,230,231,232,233]. In conclusion, there is the need to uncover the mechanisms by which these pathways coordinate the nutrient homeostasis of phosphate and lipids, as well as the fungal growth, arbuscule development and longevity.

#### 3.5.4. Sporulation

With the nourishment of plant sugars and lipids, AM fungi produce the lipid-rich vesicles (in Glomeromycotina fungi, except for the Gigasporales; see Figure 7H), or auxiliary cells (in Gigasporaceae fungi; see Figure 7K) to supply energy for the development of extraradical hyphae and the formation of new spores outside the roots (Figure 7I,J) to complete their life cycle.

AM fungal sugars and lipids can be converted into trehalose, glycogen and triacylglycerols to be used for the fungal growth and development and the storage in spores [3,181,182]. Recent studies have shown that certain exogenous fatty acids can be taken up by *R. irregularis* as an organic carbon source and stimulant to facilitate the hyphal growth and the secondary spore formation at the asymbiotic stage [234,235]. Moreover, the co-treatment of myristate, organic nitrogen and two plant hormones (both strigolactone and methyl jasmonate) can significantly induce the sporulation of *R. clarus* during asymbiotic cultures [236].

The previous studies reported that the SNF1, cAMP-PKA and TOR signaling pathways regulate sporulation in numerous species of filamentous fungi, at the cellular and molecular levels [30,185,200,237,238]. Rapamycin treatment and inactivation TOR can induce the biogenesis of lipid droplet required for triacylglycerol accumulation, whereas PKA regulates the degradation of triacylglycerol and SNF1 regulates the lipid mobilization required for fungal development and sporulation [29,92,112,185,187,200].

cAMP-PKA, Snf1, Sch9, TOR and Pho85 can control the fungal cell autophagy, essential for the pathogenicity and sporulation [239,240]. The deletion of autophagy-related gene *ATG8* leads to defects in infection, storage lipids and sporulation [202,241]. Phosphate homeostasis under the control of the PHO pathway which affects the carbon-phosphate trade between two partners and the carbon acquisition of AM fungi [204], also can regulate the sporulation of AM fungi.

The obligate biotrophic nature of AM fungi leads to the limitation of axenic culture for spore acquisition [242], which impedes their application on agricultural production. It is worth to investigate the roles of the cAMP-PKA, SNF1, TOR and PHO pathways in lipid-mediated sporulation of AM fungi during the *in planta* phase and the *in vitro* Ri T-DNA transformed roots. It would make sense to increase spore production, which is beneficial for improving the yield of crops.

## 4. Conclusions and Future Perspectives

In conclusion, we here provide the conserved cAMP-PKA, SNF1, TOR and PHO pathways in distinct AM fungi genera to generate hypotheses for further function analysis. These AM fungal nutrient signaling cascades may respond to various nutrient availabilities, including but not limited to carbon, nitrogen and phosphate, and regulate the expression of target genes involved in nutrient sensing and transport, trehalose and lipid metabolism, amino acid and ribosome biogenesis, stress responses and autophagy. These pathways with crosstalk and common targets play important roles in the spore germination, hyphal growth, sporulation in AM fungi and the appressorium formation and arbuscule longevity during AM symbiosis.

The results of the qRT-PCR analysis indicate that the key genes (e.g., *Snf1*, *Cyr1*, *Pde2*, *Bcy1*, *Tpk1*, *Tpk3*, *Tor2*, *Tip41*, *Msn2*, *Atg8*) in these pathways may participate in the regulation of appressorium formation and penetration, and they can be considered as the marker genes of the penetration at the early symbiotic stage. On the other hand, the expression patterns of *Rim15*, which encodes a serine/threonine protein kinase in AM fungi, indicate that it may serve as the arbuscule-associated marker gene at the symbiotic stage.

We thus propose a draft model that these four nutrients signaling regulates AM fungal growth at the asymbiotic stage, appressorium formation, arbuscule development and sporulation within the roots (see Figure 7L). Our findings will inspire further investigation on the roles of these nutrient signaling pathways and their crosstalk in the regulation of multiple cellular and physiological processes in AM symbiosis, but it is worth noting that only solid evidence or experiments can tell the truth about the gene functions in AM fungi.

Recent study has shown that multiple fungi can take up environmental small RNAs (sRNAs) [243] to silence fungal genes through RNA interference machinery. This is so-called spray-induced gene silencing (SIGS), which can work directly on fungal tissues to induce genes silencing during the asymbiotic, presymbiotic and symbiotic stages. It is worth noting that SIGS displays a powerful tool for studies on AM fungal gene functions involved in mutualistic plant–fungal interactions and applications on agricultural practices.

## 5. Patents

Application of the marker genes in *Rhizophagus irregularis* at the early stage of AM symbiosis. The application number is 202110785269.5.

## Figures and Tables

**Table 1:** Fungal genomes used in this study.

Phylum		Types	Fungal Genomes	References
Mucoromycota	Glomeromycotina	Arbuscular mycorrhiza	*Rhizophagus irregularis* DAOM197198	[33,34,40]
			*Rhizophagus clarus* HR1	[35]
			*Rhizophagus cerebriforme* DAOM227022	[36]
			*Gigaspora margarita* BEG34	[38]
			*Gigaspora rosea* DAOM194575	[36]
			*Diversispora epigaea* IT104	[37]
	Mucoromycotina	Animal pathogen	*Podila verticillata* NRRL 6337	GenBank: KN042421.1
			*Mucor lusitanicus* MU402	[41]
			*Rhizopus delemar* RA 99-880	[42]
Basidiomycota		Ectomycorrhizal mycorrhiza	*Hebeloma cylindrosporum* h7	[43]
			*Laccaria bicolor* S238N-H82	[44]
			*Pisolithus tinctorius* Marx 270	[43]
		Root endophyte	*Serendipita indica* DSM 11827	[45]
		Orchid mycorrhiza	*Tulasnella calospora* MUT 4182	[43]
		Plant pathogen	*Sporisorium scitamineum* SscI8	[46]
			*Ustilago maydis* 521	[47]
		Animal pathogen	*Cryptococcus neoformans* var. *neoformans* JEC21	[48]
Ascomycota		Ectomycorrhizal mycorrhiza	*Tuber melanosporum* Mel28	[49]
		Plant pathogen	*Fusarium graminearum* PH-1	[50]
			*Fusarium oxysporum* f. sp. lycopersici 4287	[51]
			*Pyricularia grisea* NI907	[52,53]
			*Colletotrichum higginsianum* IMI 349063	[54,55]
			*Zymoseptoria tritici* IPO323	[56]
			*Verticillium dahliae* VdLs.17	[57]
		Animal pathogen	*Aspergillus fumigatus* Af293	[58]
		Saprotroph or pathogen	*Neurospora crassa* OR74A	[59]

**Table 2:** The conserved components of nutrient signaling pathways in AM fungi and their transcript patterns in AM fungal tissues.

No	Homolog	Description	Accession Numbers							Conserved Domains	Expression Profile Retrieved from Transcriptome ^1^													
											*R. irregularis*							*G. rosea*						
			*S. cerevisiae*	*R. irregularis*	*R. clarus*	*G. cerebriforme*	*G. rosea*	*G. margarita*	*D. epigaea*		Spores			Mycorrhizas				Spores			Mycorrhizas			
	**Sucrose non-fermenting 1 (SNF1) protein kinase signaling pathway**																							
1	**Snf3/Hxts**	Sugar sensor or transporter	CAA98771.1	XP025173015.1	GBC07484.1	RIA98081.1	RIB05796.1	KAF0510814.1	RHZ52775.1	MFS_HXT, Sugar_tr, xylE	3.6	3.5	3.6	0.0	0.0	1.9	↓	4.0	4.0	4.0	8.2	8.7	8.6	↑
2	**Hxk1**	Hexokinase	KZV11659.1	XP025172441.1	GBB98726.1	RIA97617.1	RIB22304.1	KAF0429716.1	RHZ53334.1	COG5026	6.4	6.5	6.3	6.4	6.8	6.6		6.2	6.4	6.5	5.9	5.7	5.7	
3	**Yck1**	Casein kinase I	KZV10871.1	XP025174102.1	GBB87197.1	RIA91239.1	RIB06770.1	KAF0535241.1	RHZ66640.1	STKc_CK1_fungal	6.3	6.3	6.3	6.6	6.5	6.5		7.8	7.8	7.9	7.0	7.0	7.3	
4	**Snf1**	Carbon catabolite derepressing protein kinase	QHB07934.1	XP025181474.1	GBB86133.1	RIA96595.1	RIB06014.1	KAF0504798.1	RHZ87761.1	STKc_AMPK_alpha, AMPKA_C, UBA_SNF1_fungi	5.4	5.5	5.5	5.3	5.1	5.2		5.8	6.0	5.9	5.2	5.3	5.7	
5	**Gal83**	5′-AMP-activated protein kinase SNF1 beta subunit	KZV11779.1	XP025175162.1	GBC02790.1	RIA96007.1	RIB23376.1	KAF0492722.1		AMPKBI, AMPK1_CBM, E_set_AMPKbeta_like_N	6.1	6.2	6.1	6.6	5.5	5.6		5.7	5.6	5.6	5.1	5.4	5.5	
6	**Sak1**	Calcium/calmodulin-dependent protein kinase kinase	KZV11897.1	XP025169502.1	GBB90230.1	RIA89443.1	RIB14285.1	KAF0377796.1	RHZ56086.1	STKc_LKB1_CaMKK	5.5	5.6	5.8	5.5	6.0	6.0		5.7	5.8	5.7	5.3	5.2	4.8	
7	**Snf4**	Nuclear protein SNF4	CAA96823.1	XP025168407.1	GBB93785.1	RIA84755.1	RIB14600.1	KAF0525700.1	RHZ44230.1	CBS_euAMPK_gamma-like_repeat2, CBS_euAMPK_gamma-like_repeat1	6.0	5.9	6.0	5.8	5.9	5.5		5.2	5.1	5.1	4.1	3.7	3.3	↓
8	**Reg1**	REG1-regulatory subunit for protein phosphatase Glc7	CAA98850.1	XP025174225.1	GBB84436.1	RIA95576.1	RIB18190.1	KAF0465234.1	RHZ64075.1	DUF1752	5.3	5.5	5.6	8.0	7.8	8.6	↑	7.7	7.9	7.8	9.1	8.5	9.2	↑
9	**Rgt1**	Fungal-specific transcription factor domain-containing protein	CAA49301.1	XP025183341.1	GBB97255.1	RIA96158.1	RIB27603.1	KAF0395517.1	RHZ69529.1	GAL4, Zn_clus, Fungal_trans, fungal_TF_MHR	3.1	2.8	2.8	4.3	2.4	3.4		4.5	4.1	4.1	3.8	4.0	4.3	
10	**Gal1**	Galactokinase	CAA84962.1	XP025167943.1	GBC05682.1	RIA85840.1	RIB23881.1	KAF0523208.1	RHZ56837.1	PLN02521	3.9	3.4	3.8	5.3	5.4	6.3	↑	5.1	5.1	5.1	6.0	6.4	6.2	↑
11	**Mig1**	DNA-binding protein creA	CAA96736.1	XP025167185.1	GBB93790.1	RIA84743.1	RIB13066.1	KAF0519624.1	RHZ72138.1	COG5048 (Zn-finger)	5.0	4.8	4.0	4.6	4.6	4.4		1.2	1.5	0.0	0.0	0.0	0.0	
12	**Ssn6**	General transcriptional repressor	CAA46973.1	XP025187165.1	GBB94614.1	RIA93188.1	RIB04547.1	KAF0478375.1	RHZ44816.1	TPR	7.0	7.1	7.0	6.8	6.9	6.9		6.8	6.9	6.7	5.7	5.6	5.6	↓
13	**Tup1**	Glucose repression regulatory protein	QHB07212.1	XP025177165.1	GBB99361.1	RIA83988.1	RIB05835.1	KAF0554834.1	RHZ78296.1	WD40, Tup_N	7.8	7.9	7.8	7.6	7.7	8.3		6.7	6.8	6.8	6.3	6.1	6.0	
	**cAMP-dependent protein kinase A (cAMP-PKA) signaling pathway**																							
14	**Cdc25**	Cell division control protein 25	KZV09553.1	XP025169177.1	GBB99424.1	RIA94624.1	RIB25226.1	KAF0428185.1	RHZ74526.1	RasGEF, RasGEF_N, SH3_Sdc25	6.3	6.4	6.4	6.3	5.7	6.2	↓	6.4	6.5	6.5	7.2	7.1	7.7	↑
15	**Ras1**	Ras-like protein	CAA99298.1	XP025166744.1	GBB97044.1	RIA98161.1	RIB12382.1	KAF0440412.1	RHZ56098.1	small_GTPase	9.1	9.2	9.2	8.4	8.5	8.3	↓	3.6	3.8	3.5	3.7	2.5	2.0	
16	**Ira1**	GTPase-activator protein for Ras-like GTPase	AAA34709.1	XP025175181.1	GBC02045.1	RIA95985.1	RIB13611.1	KAF0427762.1	RHZ89446.1	RasGAP_Neurofibromin_like, CRAL_TRIO_2, RasGAP, PH-like	5.1	5.1	5.0	4.9	3.7	4.4	↓	5.5	5.7	5.5	4.2	4.1	4.4	↓
17	**Gpa3**	Guanine nucleotide-binding protein alpha subunit	QHB08090.1	XP025188707.1	GBB91367.1	RIA81730.1	RIB01335.1	KAF0408695.1	RHZ45261.1	G-alpha	6.5	6.7	6.6	5.5	6.3	6.4	↓	4.6	4.6	4.4	2.9	3.7	3.2	↓
18	**Cyr1**	Adenylate cyclase	CAA89295.1	XP025179329.1	GBB94024.1	RIA93841.1	RIB11944.1	KAF0398164.1	RHZ86430.1	PP2Cc, CYCc, RA_CYR1_like	5.3	5.3	5.4	5.8	5.0	4.8		6.0	6.1	6.0	6.2	5.7	6.2	↑
19	**Bcy1**	PKA regulatory subunit	QHB09340.1	XP025186511.1	GBC10838.1	RIA97450.1	RIB13909.1	KAF0528101.1	RHZ83631.1	CAP_ED	5.6	5.7	5.9	6.3	6.4	5.8		7.1	7.0	6.9	6.8	7.0	6.7	
20	**Tpk1**	cAMP-dependent protein kinase catalytic subunit	KZV10110.1	XP025170217.1	GBB88842.1	RIA98707.1	RIB04262.1	KAF0332898.1	RHZ57338.1	STKc_PKA_like	5.6	5.6	5.8	5.9	5.8	6.8		6.4	6.3	6.4	5.6	5.3	6.1	
21	**Pde2**	cAMP phosphodiesterase	CAA99689.1	XP025182850.1	GBC09025.1	RIA86790.1	RIB09177.1	KAF0469187.1	RHZ67223.1	PDEase_I	4.1	4.2	4.0	5.7	5.0	5.0	↑	4.5	4.6	4.9	4.0	4.8	4.9	
22	**Yak1**	YAK1-ser/thr protein kinase	CAA89437.1	XP025178972.1	GBC09926.1	RIA82219.1	RIB05244.1	KAF0349434.1	RHZ76762.1	PKc_YAK1	5.4	5.4	5.6	6.1	5.7	5.9		6.8	6.7	6.7	5.9	5.8	5.9	↓
	**Target of rapamycin (TOR) kinase signaling pathway**																							
23	**Ssy1**	Amino acid permease	QHB07619.1	XP025173052.1	GBB94153.1	RIA83029.1	RIB02084.1	KAF0517317.1	RHZ74142.1	LysP, 2A0306	3.2	3.1	2.6	8.2	7.6	8.6	↑	5.6	5.5	5.6	5.8	4.3	5.9	
24	**Mep2**	Ammonium transporter	CAA96025.1	XP025183237.1	GBC08321.1	RIA89994.1	RIB20969.1	KAF0484149.1	RHZ83241.1	AmtB	5.2	5.2	5.5	6.1	6.1	5.8	↑	8.0	7.8	7.6	3.2	3.0	3.6	↓
25	**Ure2**	Glutathione S-transferase	CAA96134.1	XP025188118.1	GBC09082.1	RIA99246.1	RIB13374.1	KAF0420358.1		GST_C_, GST_N_Ure2p_like, GstA	6.7	6.8	6.6	6.9	5.3	6.3	↓	6.1	5.9	6.1	5.9	5.4	5.5	
26	**Gat1**	Nitrogen regulatory protein areA	KZV11580.1	XP025166471.1	GBC08162.1	RIA92069.1	RIB23596.1	KAF0391989.1	RHZ89193.1	ZnF_GATA, DUF1752, GAT1	1.3	0.0	0.0	0.0	0.0	0.0		4.3	4.5	4.4	5.0	4.7	4.9	↑
27	**Gtr1**	GTP-binding protein GTR1	QHB10618.1	XP025166820.1	GBC04641.1	RIA82430.1	RIB25894.1	KAF0507139.1	RHZ45447.1	RagA_like	6.1	6.0	6.0	6.1	6.1	4.4		6.2	6.2	6.2	5.8	5.9	5.5	
28	**Gtr2**	Ras-related GTP-binding protein	BAA28781.1	XP025175556.1	GBB95986.1	RIA90228.1	RIB22850.1	KAF0500663.1	RHZ88007.1	Gtr1_RagA, RagC_like, P-loop_NTPase	6.3	6.4	6.4	6.0	6.1	6.0		6.4	6.3	6.4	6.1	5.8	5.7	
29	**Tor2**	FKBP12-rapamycin complex-associated protein	CAA50548.1	XP025188708.1	GBB93380.1	RIA95684.1	RIB03527.1	P32600.3	RHZ66605.1	FAT, TEL1, PIKKc_TOR	5.8	6.0	5.9	5.6	5.5	5.7	↓	6.2	6.2	6.1	5.3	5.0	5.5	↓
30	**Kog1**	Regulatory-associated protein of TOR	QHB09177.1	XP025188793.1	GBC02948.1	RIA93930.1	RIB12272.1	KAF0421509.1	RHZ51963.1	Raptor_N, WD40	5.6	5.8	5.6	5.7	6.1	6.2		6.6	6.4	6.4	6.4	6.4	6.6	↑
31	**Lst8**	Target-rapamycin complex subunit LST8	QHB11374.1	XP025168325.1	GBB93049.1	RIA94383.1	RIB13947.1	KAF0366345.1	RHZ52815.1	WD40	6.1	6.0	6.2	6.5	5.6	6.2		6.1	6.1	6.1	5.3	5.5	5.4	
32	**Sch9**	Serine/threonine-protein kinase	CAA40853.1	XP025179023.1	GBB99293.1	RIA97083.1	RIB30460.1	KAF0544487.1	RHZ75318.1	STKc_Sck1_like, C2 surperfamily, PKc_like	2.1	2.5	2.0	0.0	2.5	0.0		3.4	3.7	3.4	0.9	0.0	1.7	↓
33	**Tap42**	TAP42-like protein	KZV08874.1	XP025174300.1	GBC04705.1	RIA84937.1	RIB23487.1	KAF0516295.1	RHZ77344.1	TAP42	5.8	5.9	5.9	5.6	5.2	5.0	↓	6.3	6.1	6.2	5.4	5.2	5.5	↓
34	**Rrd1**	Serine/threonine-protein phosphatase 2A activator	QHB09229.1	XP025178889.1	GBC03931.1	RIA82137.1	RIB22358.1	KAF0482349.1	RHZ86199.1	PTPA	5.2	5.3	5.5	5.6	4.8	5.5		5.7	5.4	5.7	5.3	5.6	5.1	
35	**Rrd2**	Serine/threonine-protein phosphatase 2A activator	QHB12111.1	XP025179635.1	GBB93114.1	RIA87981.1	RIB08558.1	KAF0386976.1	RHZ72688.1	PTPA	6.7	6.7	6.8	6.4	6.1	6.9		5.7	5.7	5.9	6.0	5.2	5.8	
36	**Tip41**	TIP41-domain-containing protein	KZV07556.1	XP025181449.1	GBB97298.1	RIA84855.1	RIB12158.1	KAF0369945.1	RHZ86053.1	TIP41	4.7	4.5	4.7	5.0	5.2	4.7		5.6	5.5	5.6	4.5	4.0	3.5	↓
37	**Pph22**	PP2A catalytic subunit	CAA98765.1	XP025186163.1		RIA78842.1	RIB01551.1	KAF0479568.1	RHZ81746.1	MPP_PP2A_PP4_PP6	7.7	7.9	7.7	7.5	7.3	7.7		7.8	7.8	7.8	6.9	7.3	7.1	
38	**Tpd3**	Protein phosphatase PP2A regulatory subunit A	QHB06631.1	XP025181440.1	GBB86608.1	RIA96926.1	RIB23797.1	KAF0429327.1	RHZ79342.1	HEAT_2, HEAT, PRK13800	6.8	6.8	6.8	7.0	7.0	6.9		7.4	7.3	7.3	7.4	7.6	7.4	↑
39	**Cdc55**	Protein phosphatase PP2A regulatory subunit B	AAA34482.1	XP025174227.1	GBC09955.1	RIA95590.1	RIB10219.1	KAF0437825.1	RHZ64089.1	CDC55	6.8	6.9	6.9	6.9	7.0	6.9		6.9	7.0	7.0	6.7	6.7	6.4	
40	**Rts1**	Phosphatase 2A regulatory B subunit	CAA99203.1	XP025171904.1	GBB94689.1	RIA88794.1	RIB06671.1	KAF0374544.1	RHZ47872.1	B56	6.3	6.4	6.3	7.3	6.9	7.1	↑	6.3	6.4	6.2	6.2	6.0	6.2	
41	**Sap190**	SIT4 phosphatase-associated protein	CAA82100.1	XP025183818.1	GBC07712.1	RIA90499.1	RIB24656.1	KAF0417990.1	RHZ82279.1	SAPS	5.7	5.6	5.7	6.2	6.2	6.6	↑	6.3	6.2	6.2	6.0	6.0	6.2	
42	**Fpr1**	FKBP-type peptidyl-prolyl isomerase	CAA96017.1	XP025188636.1	GBC03273.1	RIA79256.1	RIB11261.1	KAF0538299.1	RHZ84133.1	FkpA, FKBP_C, FkpA	7.7	7.8	7.7	8.2	7.8	7.7		5.5	5.5	5.5	3.8	2.7	4.6	
43	**Avo1**	Stress activated MAP kinase interacting protein	KZV07803.1	XP025173883.1	GBB90387.1	RIA85683.1	RIB07950.1	KAF0552014.1	RHZ47334.1	CRIM, SIN1_PH	5.9	5.9	6.0	5.6	4.9	5.6	↓	6.2	6.2	6.2	5.5	5.0	5.7	
	**Phosphate (PHO) signalling pathway**																							
44	**Pho84**	Phosphate transporter	QHB10616.1	XP025183371.1	GBC04213.1	RIA99731.1	RIB05912.1	KAF0463572.1	RHZ61205.1	2A0109	5.7	5.8	5.7	9.6	9.1	9.4	↑	7.3	7.4	7.5	9.1	8.9	9.3	↑
45	**Pho91**	SPX domain-containing protein similar to Pho87 and Pho90	KZV08641.1	XP025171348.1	GBC10644.1	RIA85720.1	RIB09524.1	KAF0379302.1	RHZ77664.1	SLC13_permease, SPX_PHO87_PHO90_like	5.0	5.1	5.0	5.2	5.8	5.9	↑	4.9	4.7	4.9	6.0	6.7	6.4	↑
46	**Arg82**	ARG82-dual-specificity inositol polyphosphate kinase	QHB07633.1	XP025175101.1	GBC08502.1	RIA86563.1	RIB03691.1	KAF0406837.1	RHZ46527.1	IPK	3.9	4.0	4.5	4.9	5.0	4.1		6.9	6.9	7.0	6.6	6.6	6.5	
47	**Kcs1**	Inositol hexaphosphate kinase KCS1	AAB36234.1	XP025174322.1		RIA96250.1	RIB08669.1	KAF0502809.1		IPK	5.8	5.9	5.9	6.7	6.4	6.5	↑	5.5	5.5	5.3	4.9	4.8	4.6	
48	**Adk1**	Adenylate kinase	QHB07687.1	XP025181716.1	GBC08115.1	RIA88751.1	RIB29553.1	KAF0391071.1	RHZ69843.1	adk	6.4	6.6	6.4	7.1	6.4	6.3		6.4	6.4	6.5	7.4	7.9	7.7	↑
49	**Ado1**	Adenosine kinase	QHB09727.1	XP025188792.1	GBC02949.1	RIA93929.1	RIB12283.1	KAF0421504.1	RHZ51964.1	ribokinase_pfkB, adenosine_kinase	7.1	7.1	7.0	7.4	8.2	8.1	↑	7.4	7.4	7.4	7.1	7.4	6.7	
50	**Pho81**	Cyclin-dependent protein kinase inhibitor	CAA97261.1	XP025171189.1	GBB88938.1	RIA87384.1	RIB13327.1	KAF0478836.1	RHZ82191.1	SPX_PHO81_NUC-2_like, Ank_2, PI-PLCc_GDPD_SF	5.3	5.3	5.3	8.4	7.8	7.6	↑	5.5	5.5	5.4	5.7	5.2	5.2	
51	**Pho80**	Cyclin-dependent protein kinase regulator	CAA99000.1	XP025168925.1	GBC00404.1	RIA96867.1	RIB28759.1	KAF0480379.1	RHZ79637.1	CYCLIN (Cyclin box fold)	4.4	5.1	4.8	4.5	4.6	4.9		5.6	5.8	5.6	5.6	5.8	5.8	
52	**Pho85**	Negative regulator of the PHO system	CAA68773.1	XP025181480.1	GBB86139.1	RIA96589.1	RIB12090.1	KAF0377917.1		STKc_PHO85, PKc_like	7.2	7.3	7.3	6.9	7.0	7.1		5.4	5.4	5.6	5.9	6.0	6.0	↑
53	**Pho2**	Homeodomain transcription factor	CAA64906.1	XP025175623.1	GBC04742.1	RIA91171.1	RIB14816.1	KAF0560668.1	RHZ56225.1	Homeobox	5.5	5.3	5.2	5.3	7.2	5.9	↑	6.7	6.9	6.6	8.4	8.0	8.8	↑
54	**Pho4**	HLH-domain-containing protein	QHB08372.1	XP025170451.1	GBB94725.1	RIA91802.1	RIB24056.1	KAF0419854.1	RHZ63527.1	HLH, bHLHzip_USF_MITF, bHLH_SF	6.7	6.6	6.6	6.5	6.6	6.6		5.1	4.9	5.1	5.8	5.7	5.6	↑
55	**Ino80**	SNF2 family helicase/ATPase Ino80	QHB08505.1	XP025176540.1	GBB97662.1	RIA93554.1	RIB09135.1	KAF0508025.1	RHZ78787.1	HepA, SF2_C_SNF	6.8	6.9	6.8	6.6	6.5	6.7		7.7	7.8	7.7	6.8	6.7	6.7	↓
	**Common targets and relevant components**																							
56	**Rim15**	Serine threonine protein kinase	QHB08304.1	XP025183815.1	GBC07710.1		RIB15849.1	KAF0546391.1	RHZ82277.1	STKc_MAST_like, REC_hyHK_CKI1_RcsC-like	5.2	5.2	5.1	5.6	5.6	6.1	↑	6.4	6.6	6.5	5.7	5.7	6.0	
57	**14-3-3**	14-3-3 protein	CAA46959.1	XP025174150.1	GBC07263.1	RIA97653.1	RIB22291.1	KAF0420307.1	RHZ47341.1	14-3-3, BMH1	8.3	8.4	8.3	8.3	8.3	8.5		9.4	9.3	9.4	8.8	9.1	9.0	
58	**Msn2**	STE12-like C2H2 type Zn-finger transcription factor	KZV08884.1	XP025172111.1	GBC01837.1	RIA98728.1	RIB08568.1	KAF0407570.1	RHZ45449.1	COG5048 (Zn-finger), STE	3.3	2.9	1.8	0.0	0.0	0.0		4.1	4.1	4.6	6.4	6.2	6.5	↑
59	**Rph1**	Jumonji family transcription factor	KZV11945.1	XP025184487.1	GBB85389.1	RIA98904.1	RIB17285.1	KAF0402735.1		JmjC, JmjN, ePHD	3.3	3.3	3.3	3.3	3.2	3.2	↓	2.9	2.9	2.9	2.7	2.8	2.8	↓
60	**Tps2**	Glycosyltransferase	CAA98893.1	XP025178428.1	GBB84837.1	RIA80605.1	RIB18150.1	KAF0420413.1	RHZ45612.1	PRK14501	7.1	7.2	7.3	7.4	6.8	7.4		7.7	8.0	7.7	6.4	6.5	6.5	↑
61	**Nth1**	Neutral trehalase	KZV12232.1	XP025174909.1	GBC10103.1	RIA80971.1	RIB03117.1	KAF0525729.1	RHZ89787.1	Trehalase_Ca-bi, Trehalase	6.9	7.0	7.0	8.7	7.7	8.0	↑	5.8	5.7	5.8	6.8	6.3	6.5	↑
62	**Fhl1**	Fork head domain-containing protein	QHB12349.1	XP025186513.1	GBB95086.1	RIA97453.1	RIB29328.1	KAF0409680.1	RHZ62376.1	Forkhead, COG5025	5.6	5.5	5.5	6.9	6.6	6.7	↑	5.7	5.5	5.3	5.3	4.2	5.0	
63	**Maf1**	Repressor of RNA polymerase III transcription MAF1	KZV12237.1	XP025179483.1	GBC06348.1	RIA93056.1	RIB05097.1	KAF0345972.1	RHZ79416.1	Maf1	5.9	5.9	5.8	6.0	5.9	5.6		5.9	6.1	6.0	5.3	5.8	4.7	
64	**Rps13**	40S ribosomal protein S13	CAA98882.1	XP025181192.1	GBC02894.1	RIA87612.1	RIB04680.1	KAF0396453.1	RHZ52793.1	PTZ00072	8.9	9.1	9.0	8.8	8.4	8.2	↓	8.7	8.7	8.7	8.2	8.1	7.7	
65	**Gcn2**	Serine/threonine-protein kinase GCN2	AAA34636.1	XP025188225.1	GBC08433.1	RIA89287.1	RIB06418.1	KAF0497404.1	RHZ77885.1	HGTP_anticodon2, STKc_EIF2AK4_GCN2_rpt2	5.5	5.4	5.5	5.0	5.4	5.5		5.7	5.7	5.7	4.7	4.5	4.6	↓
66	**Gcn4**	Amino acid starvation-responsive transcription factor GCN4	QHB08060.1	XP025166650.1	GBB92808.1	RIA86909.1	RIB09613.1	KAF0458657.1	RHZ87741.1	bZIP_GCN4	8.4	8.2	8.6	9.6	8.8	9.3	↑	6.9	6.7	6.9	7.5	8.3	7.0	↑
67	**Atg8**	Autophagy-like protein 8	QHB06698.1	XP025175505.1	GBB98445.1	RIA88779.1	RIB13205.1	KAF0352158.1	RHZ64615.1	Ubl_ATG8	9.5	9.4	9.5	9.4	9.8	9.2		9.3	9.1	9.3	9.6	9.9	9.2	↑
68	**Ssa3**	Heat shock protein 70	CAA84896.1	XP025181914.1	GBB89555.1	RIA88208.1	RIB01431.1	KAF0462691.1	RHZ75893.1	PTZ00009, HSP70	10.5	10.7	10.5	11.0	10.8	10.6		8.6	8.7	8.7	9.8	9.9	9.4	↑
69	**Sod1**	Superoxide dismutase Cu-Zn	CAA89634.1	XP025178555.1	GBB95268.1	RIA94955.1	RIB09248.1	KAF0517010.1	RHZ44790.1	Sod_Cu	10.6	10.6	10.5	10.6	10.6	10.8		6.6	6.7	6.6	5.0	2.8	3.9	↓
70	**Sod2**	Mitochondrial Mn-superoxide dismutase	QHB08994.1	XP025168721.1	GBC00991.1	RIA91372.1	RIB04073.1	KAF0438330.1	RHZ79502.1	SodA, PLN02471	9.4	9.6	9.4	7.8	7.8	7.8	↓	8.6	8.8	8.6	7.6	7.3	7.2	↓
71	**Rho1**	GTP-binding protein rhoA	QHB12411.1	XP025181617.1	GBC08606.1	RIA85906.1	RIB06833.1	KAF0421499.1	RHZ75862.1	RhoA_like, RHO, P-loop_NTPase	9.2	9.3	9.2	9.2	9.1	9.2		6.6	6.6	6.7	8.2	8.1	8.3	↑
72	**Hog1**	Mitogen-activated protein kinase HOG1	CAA97680.1	XP025185210.1	GBB87021.1	RIA92778.1	RIB04295.1	KAF0432463.1		STKc_Sty1_Hog1	6.9	7.0	7.0	7.4	7.4	7.3		6.8	7.0	7.1	8.0	8.6	8.4	↑

^1^ The last column indicates the significant difference of the expression level in mycorrhizal roots when compared with that in the spores. The upward arrows indicate the gene expression is significantly up-regulated in mycorrhizal roots. The downward arrows indicate that the gene expression in mycorrhizal roots is significantly down-regulated. Heatmap was generated using the log_2_ (FPKM+1) transformed expression of genes found in the genomes of *R. irregularis* and *G. rosea*.

## Data Availability

The GEO accession numbers of RNA-seq reads at NCBI GEO database (https://www.ncbi.nlm.nih.gov/geo/, accessed on 1 February 2018) are as follows: germinating spores (GSM1658278, GSM1658280, GSM1658282) and mycorrhizal roots (GSM1658563, GSM1658564, GSM1658565) of *R. irregularis* and germinating spores (GSM1657757, GSM1657758, GSM1657759) and mycorrhizal roots (GSM1657861, GSM1657862, GSM1657863) of *G. rosea*.

## Supplementary Materials

The following are available online at https://www.mdpi.com/article/10.3390/microorganisms9081557/s1, Table S1: BLASTp result and detail information of putative proteins in fungal genomes, Table S2: The functions of components in nutrient signaling pathways in pathogenic and beneficial fungi, Table S3: List of the primers used in qRT-PCR. The detail information including accession numbers are shown in Supplemental Table S1.
