# Supplementary material for: Genome-Wide Analysis of Nutrient Signaling Pathways Conserved in Arbuscular Mycorrhizal Fungi
Source: Microorganisms. 2021 Jul 22;9(8):1557. doi: 10.3390/microorganisms9081557 (PMC8401276; doi:10.3390/microorganisms9081557)
Supplement: Supplementary file 1 [file microorganisms-09-01557-s001.zip › Supplemental Table S2.pdf]

Supplemental Table S2. The functions of components in nutrient signaling pathways in pathogenic and beneficial fungi.

Gene	Fungi	Description	References
**Sucrose non-fermenting 1 (SNF1) protein kinase signaling pathway**			
Hxk1	*F. verticillioides*	*HXK1* regulated carbon catabolism, sporulation, and pathogenesis.	[1]
	F. graminearum	The deletion led to inhibited vegetative growth and conidiation, whereas the overexpression led to increased deoxynivalenol production.	[2]
	Botrytis cinerea	The deletion mutants of *HXK1* were defective in growth and virulence.	[3]
Snf1	*M. oryzae*	The knock-out mutants displayed a defect in growth, conidial germination, appressorium formation, pathogenicity and sporulation.	[4]
	F. oxysporum	The disruption led to defects in the suppression of cell wall-degrading enzymes, utilization of certain carbon sources, and virulence.	[5]
	V. dahliae	The disruption of *VdSNF1* led to defects in growth, virulence, and colonization.	[6]
	Pestalotiopsis microspora	The deletion mutants contributed to defects in vegetative growth, pigmentation, conidia, cell wall integrity.	[7]
	Alternaria alternata	The deletion mutants were defective in in aerial mycelium growth, conidia, and germination with smaller lesions on detached citrus leaves, and stronger tolerance to some stressors.	[8]
Gal83	*M. oryzae*	The mutants of *ΔMosnf4*, *ΔMosak1*, *ΔMosnf1*, *ΔMosip2*, and *ΔMosak1ΔMotos3*, displayed distinct defects in lipid mobilization, sporulation, conidial germination, and appressorium formation.	[9]
Sak1			
Snf4			
Reg1	*B. cinerea*	The gene knock-out mutant of *bcreg1* kept the ability to penetration but were defective in pathogenicity and conidia formation.	[10]
Ssn6	*U. maydis*	The homologue of *Ssn6*, *Sql1* alleles in a certain form induced the fungal filamentous growth which can be reversed by exogenous cAMP.	[11]
Tup1	*U. maydis*	The deletion of the *Umtup1* led to defects in the mating, filamentation and virulence.	[12]
	M. oryzae	The disruption of *MoTUP1* suppressed vegetative growth and conidiogenesis, and led to the formation of appressorium-like structures that cannot penetrate into the host cells.	[13]
**cAMP-dependent protein kinase A (cAMP-PKA) signaling pathway**			
Cdc25	*C. higginsianum*	The deletion of *ChCDC25* led to defects in vegetative growth, conidiation, conidium germination, appressorium formation, stress chemicals tolerance, and virulence on *Arabidopsis* leaves.	[14]
	U. maydis	The deletion mutants of CDC25-like gene *sql2* were defective in pathogenic development, whereas the overexpression mutants of *sql2* were shown an enhanced filamentous growth that cannot be repressed by exogenous cAMP.	[15]
Ras1	*M. oryzae*	The overactive *MoRas2* resulted in improper activation of these two cascades, leading to abnormal appressorium formation without surface attachment.	[16]
	C. orbiculare	The disrupted mutants of *coras2* were defective in conidial germination and pathogenesis to the cucumber cotyledons.	[17]
Ira1		CoIra1 and CoRas2 colocalized in a vesicle-like structure in the appressorium, and the mutants of *coira1* had a defective in infection morphogenesis on cucumber plants.	
Gpa3	*M. grisea*	The disruption of Gα subunit genes *magB* restrained vegetative growth, conidiation, appressorium formation, infection, mating, and pathogenicity.	[18]

Supplemental Table S2. The functions of components in nutrient signaling pathways in pathogenic and beneficial fungi (*continued*).

Gene	Fungi	Description	References
Gpa3	*U. maydis*	The disruption of *gpa3* led to the loss of ability to respond to pheromone and defect in mating, whereas the active allele of *gpa3* resulted in mating without pheromone.	[19]
		The exogenous cAMP was comparable to pheromone to stimulate pheromone gene expression, and rescue the sterility and mutant morphology of *gpa3* deletion strains.	[20]
	S. scitamineum	Gpa3, adenylyl cyclase UAC1 and PKA catalytic subunit ADR1 are essential for proper mating, filamentation, and virulence. These genes can regulate the expression of genes encoding ROS (reactive oxygen species) catabolic enzymes in *S. scitamineum*.	[21]
	Umbilicaria muhlenbergii	The deletion of Gα subunits *UmGPA3* displayed little effects on pseudohyphal growth, the *ΔUmgpa3* mutant had defects in yeast-to-pseudohypha transition. The dominant active mutations of *UmGPA3* led to improper pseudohyphal growth and disruption of the symbiotic interaction with *Trebouxia jamesii.*	[22]
	M. oryzae	Rgs7 couples with Gα subunit MagA to sense external signals and activate cAMP signaling required for appressorium function.	[23]
Cyr1	*S. sclerotiorum*	deletion of adenylate cyclase *sac1* gene led to low cAMP levels, more microconidia, attenuated virulence and slow growth rate.	[24]
	C. higginsianum	The mutants lacking of adenylate cyclase ChAC and PKA catalytic subunits ChPKA1 displayed distinct decrease in hyphal growth, conidiation, appressorial formation, pathogenicity, and stress tolerance to cell wall inhibitors.	[25]
Bcy1	*M. circinelloides*	The four PKA regulatory subunits encoding gene *pkaR1*, *pkaR2*, *pkaR3*, and *pkaR4* with different expression levels were shown with different roles in growth, germination, cell volume, sporulation, and differentiation in each isoform mutants.	[26,27]
	C. lagenarium	The breakdown of glycogen and lipid was very rapid in PKA regulatory subunit *mac1 sum1-99* mutant, whereas the dissolution was delayed significantly in catalytic subunit cpkA mutant.	[28]
		The mutants of RPK1, showed high PKA activity that suppressed vegetative growth, conidiation, and appressorium function.	[29]
		The knockout mutants of the adenylate cyclase CAC1 and PKA catalytic subunit CPK1 germinated poorly, and form appressoria which are nonfunctional that leads to the infection defect on cucumber.	[30]
Tpk1	*M. grisea*	PKA catalytic subunit *cpkA* was required for appressorial penetration and pathogenesis.	[31,32]
	N. crassa	The catalytic subunit pkac-1 mutants showed defects in aerial hyphae formation and conidiation.	[33]
	V. dahliae	The mutants lacking for PKA catalytic subunit genes displayed less ethylene biosynthesis and defects in conidia production.	[34]
	F. oxysporum	The mutants lacking for PKA catalytic subunit gene *FoCPKA* lost virulence and showed a decrease in vegetative growth, spore production, root penetration and vascular colonization on *A. thaliana* roots, and the distance between septas of *focpkA* mutants was longer than that of wild-type strain.	[35]
	M. oryzae	The PKA catalytic subunits genes *Cpk2* and *CpkA* shared largely redundant functions in regulation of hyphal growth, conidiation and appressorium formation, but also played different roles during pathogenesis and morphogenesis.	[36]

Supplemental Table S2. The functions of components in nutrient signaling pathways in pathogenic and beneficial fungi (*continued*).

Gene	Fungi	Description	References
Pde2	*M. oryzae*	The loss of *PDEH* enhanced conidiation and increased intracellular cAMP levels during vegetative growth, leading to precocious appressorial development, loss of surface dependency and decrease of colonization.	[37]
		The disruption of *PDEH* can partially rescue the abnormal phenotype of G alpha subunit encoding gene *ΔmagB* mutant and PKA catalytic subunit encoding gene *Δpka1* mutant.	[38].
		MoPdeH can function upstream of the MAP kinase pathway to regulate the cell wall integrity (CWI), and also can mediate the crosstalk between cAMP pathway and osmotic sensing high osmolarity glycerol (HOG) pathway.	[39]
Yak1	*M. oryzae*	The disruption of *MoYAK1* led to defects in germination, aerial hyphal formation, conidiation, glycogen and lipid metabolism, turgor generation, penetration and cell wall integrity.	[40]
	B. cinerea	The deletion of *Yak1* resulted in lower pathogenicity, conidiation, sclerotium formation, and more sensitive to H_2O_2.	[41]
**Target of rapamycin (TOR) kinase signaling pathway**			
Gtr1	*Candida albicans*	TOR can response to phosphate, and the overexpression of *Gtr1* suppresses TORC1 signaling defects.	[42]
Tip41	*F. graminearum*	The FgFkbp12, FgPp2A, FgSit4, FgPpg1, FgTap42, FgTip41, FgAreA in TOR pathway formed a regulatory framework to regulating vegetative differentiation and virulence. FgTip41 regulated hyphal growth and virulence.	[43].
Tor2	*F. graminearum*	The deletion of *FgTor* may be lethal, as the mutants cannot be generated.	[43,44]
	M. oryzae	MoTor regulated the expression of *MoSNT2*, which stimulated the expression of autophagy genes *MoATG6/15/16/22* to regulate the conidiation, stress tolerance, cell wall integrity, and pathogenicity.	[45]
		Asd4 regulated intracellular glutamine concentration to modulate the TOR inhibition of appressorium formation, and TOR signaling was activated in the *Δasd4* deletion mutants that fail to form appressoria.	[46]
		Glucose can mediate TOR via the carbon-responsive Ampkβ subunit-like protein, ABL1. The *ΔMoabl1* mutants produced few appressoria, and fail in autophagy, and this can be restored by inactivated TOR.	[47]
Sch9	*F. graminearum*	FgSch9 interacted with FgMaf1, FgTor and FgHog1 to regulate fungal vegetative development, multiple stress responses and secondary metabolism.	[48].
Tap42 / ***Rrd2***	*F. graminearum*	Antofine treatment led to the disruption of the FgRRD2-FgTap42 which was required for pathogenicity.	[49]
Pph22/ Ppg1/ Sit4	*F. graminearum*	The deletion mutants of *FgHLTF1*, whose expression was down-regulated by *FgPPG1* deletion or rapamycin treatment, showed defects in the vegetative growth, sexual reproduction, and virulence.	[50]
		FgPpg1/Sit4 were need for inducing lipid droplets biogenesis which regulated the fungal vegetative growth, sexual development, and virulence via Nem1/Spo7-Pah1 cascade.	[51]
	M. oryzae	MoPpe1, orthologue of Sit4/Ppe1 in yeast, interacted with MoSap1 under controlled by MoTap42 and MoTip41 to regulate the fungal autophagy, vegetative growth, appressorium function, infection, and rapamycin sensitivity.	[52,53]

Supplemental Table S2. The functions of components in nutrient signaling pathways in pathogenic and beneficial fungi (*continued*).

Gene	Fungi	Description	References
**Phosphate (PHO) signaling pathway**			
Pho84	*G. margarita*	The knockdown of phosphate transceptor gene *GigmPT* (*Pho84* in yeast) by the host-induced gene silencing (HIGS) strategy led to a defect in arbuscule development.	[54]
Arg82	*Cryptococcus neoformans*	The mutants of *Arg1*, the orthologue of *Arg82* in yeast, displayed defects in the capsule production, cell wall organization, thermotolerance, cell separation, and mating filaments formation.	[55,56]
Pho81	*N. crassa*	In the mutant of *nuc-2* (*Pho81* in yeast), some genes involved in the initiation of mRNA translation were up-regulated.	[57]
	V. dahliae	The phosphate-starved *VdNUC-2* knockout mutants showed defects in radial growth, conidia production, and become more sensitive to hydrogen peroxide stress that cannot be restored by phosphate supply.	[58]
	C. neoformans	The inositol pyrophosphate IP_7 promoted virulence, and destruction of IP_7-Pho81 or repression of Pho81 causes a defect in the virulence.	[59]
Pho80	*A. fumigatus*	The *PHO80* mutant displayed a polar growth defect and the increases of vacuolar accumulation, acid phosphatase activity and phosphate concentration.	[60]
	S. indica	The expression level of *SiPho80* was upregulated to maintain phosphate homeostasis under high phosphate levels, and *SiPho80* can restore the fungal tolerance to salt and heavy metal (e.g. copper, zinc, cobalt) in yeast *Δpho80* mutant.	[61]
Pho85	*U. maydis*	The mutants of *Cdk5/Pho85* displayed defects in the morphogenesis and polar growth required for virulence.	[62,63]
Pho4	*N. crassa*	The subcellular location of NUC-1 (Pho4 in yeast) is important for regulating gene expression	[64]
Ino80	*Trichoderma virens*	IPA-1, a protein belongs to the SNF2 family, played an important role in plant disease resistance, and the deletion of *IPA-1* led to the diminished antibiotic activity of plant against *Rhizoctonia solani.*	[65]
**Common targets and relevant components**			
14-3-3	*F. mosseae*	*Fm201*, *Ri14-3-3* and *RiBMH2* restored the lethal yeast *bmh1 bmh2* double mutant. The gene silencing of both *Ri14-3-3* and *RiBMH2* impaired the arbuscule formation.	[66]
	R. irregularis		
Rim15	*Cochliobolus heterostrophus*	Deletion of *RIM15* affected the fungal pigmentation and vegetative growth.	[67]
Atg8	*M. grisea*	The *ΔMgATG8* mutants were defective in the autophagy, and failed in conidial cell death which is necessary for infection and pathogenicity.	[68]
	F. graminearum	The *ΔFgatg8* deletion mutants were defective in autophagy required for sporulation and sexual reproduction, and displayed the aerial hyphal collapse with more lipid droplets.	[69]
		The degradation of Gcn5 induced by rapamycin can suppress the cellular relocalization and acetylation of Atg8, leading to autophagy.	[70]
Rho1	*U. maydis*	The Rho1 controled cell separation and polarity, and it interacted with the high-affinity ammonium transporter Ump2 to control filamentation which was required for fungal development and pathogenesis.	[71,72]

1. Kim, H.; Smith, J.E.; Ridenour, J.B.; Woloshuk, C.P.; Bluhm, B.H. *HXK1* regulates carbon catabolism, sporulation, fumonisin B_1 production and pathogenesis in *Fusarium verticillioides*. *Microbiology* **2011**, *157*, 2658-2669, doi:10.1099/mic.0.052506-0.
2. Zhang, L.; Li, B.; Zhang, Y.; Jia, X.; Zhou, M. Hexokinase plays a critical role in deoxynivalenol (DON) production and fungal development in *Fusarium graminearum*. *Molecular Plant Pathology* **2016**, *17*, 16-28, doi:10.1111/mpp.12258.
3. Rui, O.; Hahn, M. The *Botrytis cinerea* hexokinase, Hxk1, but not the glucokinase, Glk1, is required for normal growth and sugar metabolism, and for pathogenicity on fruits. *Microbiology* **2007**, *153*, 2791-2802, doi:10.1099/mic.0.2007/006338-0.
4. Yi, M.; Park, J.H.; Ahn, J.H.; Lee, Y.H. *MoSNF1* regulates sporulation and pathogenicity in the rice blast fungus *Magnaporthe oryzae*. *Fungal Genetics and Biology* **2008**, *45*, 1172-1181, doi:10.1016/j.fgb.2008.05.003.
5. Ospina-Giraldo, M.D.; Mullins, E.; Kang, S. Loss of function of the *Fusarium oxysporum SNF1* gene reduces virulence on cabbage and *Arabidopsis*. *Current Genetics* **2003**, *44*, 49-57, doi:10.1007/s00294-003-0419-y.
6. Tzima, A.K.; Paplomatas, E.J.; Rauyaree, P.; Ospina-Giraldo, M.D.; Kang, S. *VdSNF1*, the sucrose nonfermenting protein kinase gene of *Verticillium dahliae*, is required for virulence and expression of genes involved in cell-wall degradation. *Molecular plant-microbe interactions* **2011**, *24*, 129–142, doi: 10.1094/MPMI-09-09-0217.
7. Wang, D.; Li, Y.; Wang, H.; Wei, D.; Akhberdi, O.; Liu, Y.; Xiang, B.; Hao, X.; Zhu, X. The AMP-activated protein kinase homolog Snf1 concerts carbon utilization, conidia production and the biosynthesis of secondary metabolites in the taxol-producer *Pestalotiopsis microspora*. *Genes* **2018**, *9*, 59, doi:10.3390/genes9020059.
8. Tang, K.; Lv, W.; Zhang, Q.; Zhou, C. Coding the alpha-subunit of SNF1 kinase, *Snf1* is required for the conidiogenesis and pathogenicity of the *Alternaria alternata* tangerine pathotype. *Fungal Biology* **2020**, *124*, 562-570, doi:10.1016/j.funbio.2020.02.008.
9. Zeng, X.Q.; Chen, G.Q.; Liu, X.H.; Dong, B.; Shi, H.B.; Lu, J.P.; Lin, F. Crosstalk between SNF1 pathway and the peroxisome-mediated lipid metabolism in *Magnaporthe oryzae*. *PLoS One* **2014**, *9*, e103124, doi:10.1371/journal.pone.0103124.
10. Michielse, C.B.; Becker, M.; Heller, J.; Moraga, J.; Collado, I.G.; Tudzynski, P. The *Botrytis cinerea* Reg1 protein, a putative transcriptional regulator, is required for pathogenicity, conidiogenesis, and the production of secondary metabolites. *Molecular plant-microbe interactions* **2011**, *24*, 1074-1085, doi:10.1094/MPMI-01-11-0007.
11. Loubradou, G.; Brachmann, A.; Feldbrügge, M.; Kahmann, R. A homologue of the transcriptional repressor Ssn6p antagonizes cAMP signalling in *Ustilago maydis*. *Molecular microbiology* **2001**, *40*, 719–730, doi:10.1046/j.1365-2958.2001.02424.x.
12. Elias-Villalobos, A.; Fernandez-Alvarez, A.; Ibeas, J.I. The general transcriptional repressor Tup1 is required for dimorphism and virulence in a fungal plant pathogen. *PLoS Pathogens* **2011**, *7*, e1002235, doi:10.1371/journal.ppat.1002235.
13. Chen, Y.; Zhai, S.; Sun, Y.; Li, M.; Dong, Y.; Wang, X.; Zhang, H.; Zheng, X.; Wang, P.; Zhang, Z. MoTup1 is required for growth, conidiogenesis and pathogenicity of *Magnaporthe oryzae*. *Molecular Plant Pathology* **2015**, *16*, 799-810, doi:10.1111/mpp.12235.
14. Yan, Y.; Tang, J.; Yuan, Q.; Gu, Q.; Liu, H.; Huang, J.; Hsiang, T.; Zheng, L. ChCDC25 regulates infection-related morphogenesis and pathogenicity of the crucifer anthracnose fungus *Colletotrichum higginsianum*. *Frontiers in Microbiology* **2020**, *11*, 763, doi:10.3389/fmicb.2020.00763.

15. Muller, P.; Katzenberger, J.D.; Loubradou, G.; Kahmann, R. Guanyl nucleotide exchange factor Sql2 and Ras2 regulate filamentous growth in *Ustilago maydis*. *Eukaryot Cell* **2003**, *2*, 609-617, doi:10.1128/ec.2.3.609-617.2003.
16. Zhou, X.; Zhao, X.; Xue, C.; Dai, Y.; Xu, J.R. Bypassing both surface attachment and surface recognition requirements for appressorium formation by overactive ras signaling in *Magnaporthe oryzae*. *Molecular Plant Microbe Interact* **2014**, *27*, 996-1004, doi:10.1094/MPMI-02-14-0052-R.
17. Harata, K.; Kubo, Y. Ras GTPase activating protein CoIra1 is involved in infection-related morphogenesis by regulating cAMP and MAPK signaling pathways through CoRas2 in *Colletotrichum orbiculare*. *PLoS One* **2014**, *9*, e109045, doi:10.1371/journal.pone.0109045.
18. Liu, S.; Dean, R.A. G protein alpha subunit genes control growth, development, and pathogenicity of *Magnaporthe grisea*. *Molecular plant-microbe interactions* **1997**, *10*, 1075–1086, doi:10.1094/MPMI.1997.10.9.1075.
19. Regenfelder, E.; Spellig, T.; Hartmann, A.; Lauenstein, S.; Bölker, M.; Kahmann, R. G proteins in *Ustilago maydis*: transmission of multiple signals? *The EMBO journal* **1997**, *16*, 1934–1942, doi:10.1093/emboj/16.8.1934.
20. Krüger, J.; Loubradou, G.; Regenfelder, E.; Hartmann, A.; Kahmann, R. Crosstalk between cAMP and pheromone signalling pathways in *Ustilago maydis*. *Molecular and general genetics* **1998**, *260*, 193–198, doi:10.1007/s004380050885.
21. Chang, C.; Cai, E.; Deng, Y.Z.; Mei, D.; Qiu, S.; Chen, B.; Zhang, L.; Jiang, Z. cAMP/PKA signalling pathway regulates redox homeostasis essential for *Sporisorium scitamineum* mating/filamentation and virulence. *Environmental Microbiology* **2019**, *21*, 959-971, doi:10.1111/1462-2920.14496.
22. Wang, Y.; Wei, X.; Bian, Z.; Wei, J.; Xu, J.R. Coregulation of dimorphism and symbiosis by cyclic AMP signaling in the lichenized fungus *Umbilicaria muhlenbergii*. *PNAS* **2020**, doi:10.1073/pnas.2005109117.
23. Li, X.; Zhong, K.; Yin, Z.; Hu, J.; Wang, W.; Li, L.; Zhang, H.; Zheng, X.; Wang, P.; Zhang, Z. The seven transmembrane domain protein MoRgs7 functions in surface perception and undergoes coronin MoCrn1-dependent endocytosis in complex with Galpha subunit MoMagA to promote cAMP signaling and appressorium formation in *Magnaporthe oryzae*. *PLoS Pathogens* **2019**, *15*, e1007382, doi:10.1371/journal.ppat.1007382.
24. Jurick, W.M., 2nd; Rollins, J.A. Deletion of the adenylate cyclase (*sac1*) gene affects multiple developmental pathways and pathogenicity in *Sclerotinia sclerotiorum*. *Fungal Genetics and Biology* **2007**, *44*, 521-530, doi:10.1016/j.fgb.2006.11.005.
25. Zhu, W.; Zhou, M.; Xiong, Z.; Peng, F.; Wei, W. The cAMP-PKA signaling pathway regulates pathogenicity, hyphal growth, appressorial formation, conidiation, and stress tolerance in *Colletotrichum higginsianu*. *Frontiers in Microbiology* **2017**, *8*, 1416, doi:10.3389/fmicb.2017.01416.
26. Ocampo, J.; Fernandez Nunez, L.; Silva, F.; Pereyra, E.; Moreno, S.; Garre, V.; Rossi, S. A subunit of protein kinase a regulates growth and differentiation in the fungus *Mucor circinelloides*. *Eukaryot Cell* **2009**, *8*, 933-944, doi:10.1128/EC.00026-09.
27. Ocampo, J.; McCormack, B.; Navarro, E.; Moreno, S.; Garre, V.; Rossi, S. Protein kinase A regulatory subunit isoforms regulate growth and differentiation in *Mucor circinelloides*: essential role of PKAR4. *Eukaryot Cell* **2012**, *11*, 989-1002, doi:10.1128/EC.00017-12.

28. Thines, E.; Weber, R.W.; Talbot, N.J. MAP kinase and protein kinase A-dependent mobilization of triacylglycerol and glycogen during appressorium turgor generation by *Magnaporthe grisea*. *The Plant cell* **2000**, *12*, 1703–1718, doi:10.1105/tpc.12.9.1703.
29. Takano, Y.; Komeda, K.; Kojima, K.; Okuno, T. Proper regulation of cyclic AMP-dependent protein kinase is required for growth, conidiation, and appressorium function in the anthracnose fungus *Colletotrichum lagenarium*. *Molecular Plant Microbe Interact* **2001**, *14*, 1149-1157.
30. Yamauchi, J.; Takayanagi, N.; Komeda, K.; Takano, Y.; Okuno, T. cAMP-PKA signaling regulates multiple steps of fungal infection cooperatively with Cmk1 MAP kinase in *Colletotrichum lagenarium*. *Molecular Plant-Microbe Interactions* **2004**, *17*, 1355-1365, doi:10.1094/mpmi.2004.17.12.1355.
31. Mitchell, T.K.; Dean, R.A. The cAMP-dependent pmtein kinase catalytic subunit is required for appressorium formation and pathogenesis by the rice blast pathogen *Magnaporthe grisea*. *The Plant Cell* **1995**.
32. Xu, J.R.; Urban, M.; Sweigard, J.A.; Hamer, J.E. The *CPKA* gene of *Magnaporthe grisea* is essential for appressorial penetration. *Molecular Plant-Microbe Interactions* **1997**, *10*, 187-194.
33. Banno, S.; Ochiai, N.; Noguchi, R.; Kimura, M.; Yamaguchi, I.; Kanzaki, S.; Murayama, T.; Fujimura, M. A catalytic subunit of cyclic AMP-dependent protein kinase, PKAC-1, regulates asexual differentiation in *Neurospora crassa*. *Genes & genetic systems* **2005**, *80*, 25–34, doi:10.1266/ggs.80.25.
34. Tzima, A.; Paplomatas, E.J.; Rauyaree, P.; Kang, S. Roles of the catalytic subunit of cAMP-dependent protein kinase A in virulence and development of the soilborne plant pathogen *Verticillium dahliae*. *Fungal Genetics and Biology* **2010**, *47*, 406-415, doi:10.1016/j.fgb.2010.01.007.
35. Kim, H.S.; Park, S.Y.; Lee, S.; Adams, E.L.; Czymmek, K.; Kang, S. Loss of cAMP-dependent Protein Kinase A affects multiple traits important for root pathogenesis by *Fusarium oxysporum*. *Molecular Plant-Microbe Interactions* **2011**.
36. Selvaraj, P.; Shen, Q.; Yang, F.; Naqvi, N.I. Cpk2, a catalytic subunit of cyclic AMP-PKA, regulates growth and pathogenesis in rice blast. *Frontiers in Microbiology* **2017**, *8*, doi:10.3389/fmicb.2017.02289.
37. Ramanujam, R.; Naqvi, N.I. PdeH, a high-affinity cAMP phosphodiesterase, is a key regulator of asexual and pathogenic differentiation in *Magnaporthe oryzae*. *PLoS Pathogens* **2010**, *6*, e1000897, doi:10.1371/journal.ppat.1000897.
38. Zhang, H.; Liu, K.; Zhang, X.; Tang, W.; Wang, J.; Guo, M.; Zhao, Q.; Zheng, X.; Wang, P.; Zhang, Z. Two phosphodiesterase genes, *PDEL* and *PDEH*, regulate development and pathogenicity by modulating intracellular cyclic AMP levels in *Magnaporthe oryzae*. *PLoS One* **2011**, *6*, e17241, doi:10.1371/journal.pone.0017241.
39. Yin, Z.; Tang, W.; Wang, J.; Liu, X.; Yang, L.; Gao, C.; Zhang, J.; Zhang, H.; Zheng, X.; Wang, P.; et al. Phosphodiesterase MoPdeH targets MoMck1 of the conserved mitogen-activated protein (MAP) kinase signalling pathway to regulate cell wall integrity in rice blast fungus *Magnaporthe oryzae*. *Molecular Plant Pathology* **2016**, *17*, 654-668, doi:10.1111/mpp.12317.
40. Han, J.H.; Lee, H.M.; Shin, J.H.; Lee, Y.H.; Kim, K.S. Role of the MoYAK1 protein kinase gene in *Magnaporthe oryzae* development and pathogenicity. *Environmental Microbiology* **2015**, *17*, 4672-4689, doi:10.1111/1462-2920.13010.

41. Yang, Q.; Zhang, J.; Hu, J.; Wang, X.; Lv, B.; Liang, W. Involvement of BcYak1 in the regulation of vegetative differentiation and adaptation to oxidative stress of *Botrytis cinerea*. *Frontiers in Microbiology* **2018**, *9*, 281, doi:10.3389/fmicb.2018.00281.
42. Liu, N.-N.; Flanagan, P.R.; Zeng, J.; Jani, N.M.; Cardenas, M.E.; Moran, G.P.; Köhler, J.R. Phosphate is the third nutrient monitored by TOR in *Candida albicans* and provides a target for fungal-specific indirect TOR inhibition. *PNAS* **2017**, *114*, 6346-6351, doi:10.1073/pnas.1617799114.
43. Yu, F.; Gu, Q.; Yun, Y.; Yin, Y.; Xu, J.R.; Shim, W.B.; Ma, Z. The TOR signaling pathway regulates vegetative development and virulence in *Fusarium graminearum*. *New Phytologist* **2014**, *203*, 219-232, doi:10.1111/nph.12776.
44. Wang, C.; Zhang, S.; Hou, R.; Zhao, Z.; Zheng, Q.; Xu, Q.; Zheng, D.; Wang, G.; Liu, H.; Gao, X.; et al. Functional analysis of the kinome of the wheat scab fungus *Fusarium graminearum*. *PLoS Pathogens* **2011**, *7*, e1002460, doi:10.1371/journal.ppat.1002460.
45. He, M.; Xu, Y.; Chen, J.; Luo, Y.; Lv, Y.; Su, J.; Kershaw, M.J.; Li, W.; Wang, J.; Yin, J.; et al. MoSnt2-dependent deacetylation of histone H3 mediates MoTor-dependent autophagy and plant infection by the rice blast fungus *Magnaporthe oryzae*. *Autophagy* **2018**, *14*, 1543-1561, doi:10.1080/15548627.2018.1458171.
46. Marroquin-Guzman, M.; Wilson, R.A. GATA-dependent glutaminolysis drives appressorium formation in *Magnaporthe oryzae* by suppressing TOR inhibition of cAMP/PKA signaling. *PLoS Pathogens* **2015**, *11*, doi:10.1371/journal.ppat.1004851.
47. Marroquin-Guzman, M.; Sun, G.; Wilson, R.A. Glucose-ABL1-TOR signaling modulates cell cycle tuning to control terminal appressorial cell differentiation. *PLoS Genetics* **2017**, *13*, e1006557, doi:10.1371/journal.pgen.1006557.
48. Gu, Q.; Zhang, C.; Yu, F.; Yin, Y.; Shim, W.B.; Ma, Z. Protein kinase FgSch9 serves as a mediator of the target of rapamycin and high osmolarity glycerol pathways and regulates multiple stress responses and secondary metabolism in *Fusarium graminearum*. *Environmental Microbiology* **2015**, *17*, 2661-2676, doi:10.1111/1462-2920.12522.
49. Mogg, C.; Bonner, C.; Wang, L.; Schernthaner, J.; Smith, M.; Desveaux, D.; Subramaniam, R. Genomic identification of the TOR signaling pathway as a target of the plant Alkaloid Antofine in the phytopathogen *Fusarium graminearum*. *mBio* **2019**, *10*, doi:10.1128/mBio.00792-19.
50. Lv, W.; Wu, J.; Xu, Z.; Dai, H.; Ma, Z.; Wang, Z. The putative histone-like transcription factor FgHltf1 is required for vegetative growth, sexual reproduction, and virulence in *Fusarium graminearum*. *Current Genetics* **2019**, *65*, 981-994, doi:10.1007/s00294-019-00953-3.
51. Liu, N.; Yun, Y.; Yin, Y.; Hahn, M.; Ma, Z.; Chen, Y. Lipid droplet biogenesis regulated by the FgNem1/Spo7-FgPah1 phosphatase cascade plays critical roles in fungal development and virulence in *Fusarium graminearum*. *New Phytologist* **2019**, *223*, 412-429, doi:10.1111/nph.15748.
52. Qian, B.; Liu, X.; Ye, Z.; Zhou, Q.; Liu, P.; Yin, Z.; Wang, W.; Zheng, X.; Zhang, H.; Zhang, Z. Phosphatase-associated protein MoTip41 interacts with the phosphatase MoPpe1 to mediate crosstalk between TOR and cell wall integrity signaling during infection by the rice blast fungus *Magnaporthe oryzae*. *Environmental Microbiology* **2020**, doi:10.1111/1462-2920.15136.

53. Qian, B.; Liu, X.; Jia, J.; Cai, Y.; Chen, C.; Zhang, H.; Zheng, X.; Wang, P.; Zhang, Z. MoPpe1 partners with MoSap1 to mediate TOR and cell wall integrity signalling in growth and pathogenicity of the rice blast fungus *Magnaporthe oryzae*. *Environmental Microbiology* **2018**, *20*, 3964-3979, doi:10.1111/1462-2920.14421.
54. Xie, X.; Lin, H.; Peng, X.; Xu, C.; Sun, Z.; Jiang, K.; Huang, A.; Wu, X.; Tang, N.; Salvioli, A.; et al. Arbuscular mycorrhizal symbiosis requires a phosphate transceptor in the *Gigaspora margarita* fungal symbiont. *Molecular Plant Pathology* **2016**, *9*, 1583-1608, doi:10.1016/j.molp.2016.08.011.
55. Li, C.; Lev, S.; Desmarini, D.; Kaufman-Francis, K.; Saiardi, A.; Silva, A.P.G.; Mackay, J.P.; Thompson, P.E.; Sorrell, T.C.; Djordjevic, J.T. IP3-4 kinase Arg1 regulates cell wall homeostasis and surface architecture to promote clearance of *Cryptococcus neoformans* infection in a mouse model. *Virulence* **2017**, *8*, 1833-1848, doi:10.1080/21505594.2017.1385692.
56. Lev, S.; Desmarini, D.; Li, C.; Chayakulkeeree, M.; Traven, A.; Sorrell, T.C.; Djordjevic, J.T. Phospholipase C of *Cryptococcus neoformans* regulates homeostasis and virulence by providing inositol trisphosphate as a substrate for Arg1 kinase. *Infection and Immunity* **2013**, *81*, 1245-1255, doi:10.1128/IAI.01421-12.
57. Gras, D.E.; Silveira, H.C.; Martinez-Rossi, N.M.; Rossi, A. Identification of genes displaying differential expression in the *nuc-2* mutant strain of the mold *Neurospora crassa* grown under phosphate starvation. *FEMS Microbiology Letters* **2007**, *269*, 196-200, doi:10.1111/j.1574-6968.2006.00613.x.
58. Wilson, R.A.; Deng, S.; Wang, C.-y.; Zhang, X.; Wang, Q.; Lin, L. VdNUC-2, the key regulator of phosphate responsive signaling pathway, is required for *Verticillium dahliae* infection. *PLoS One* **2015**, *10*, doi:10.1371/journal.pone.0145190.
59. Desmarini, D.; Lev, S.; Furkert, D.; Crossett, B.; Saiardi, A.; Kaufman-Francis, K.; Li, C.; Sorrell, T.C.; Wilkinson-White, L.; Matthews, J.; et al. IP_7-SPX domain interaction controls fungal virulence by stabilizing phosphate signaling machinery. *mBio* **2020**, *11*, doi:10.1128/mBio.01920-20.
60. de Gouvêa, P.F.; Soriani, F.M.; Malavazi, I.; Savoldi, M.; Goldman, M.H.d.S.; Loss, O.; Bignell, E.; da Silva Ferreira, M.E.; Goldman, G.H. Functional characterization of the *Aspergillus fumigatus PHO80* homologue. *Fungal Genetics and Biology* **2008**, *45*, 1135-1146, doi:10.1016/j.fgb.2008.04.001.
61. Loha, A.; Kashyap, A.K.; Sharma, P. A putative cyclin, *SiPHO80* from root endophytic fungus *Serendipita indica* regulates phosphate homeostasis, salinity and heavy metal toxicity tolerance. *Biochem Biophys Res Commun* **2018**, *507*, 414-419, doi:10.1016/j.bbrc.2018.11.053.
62. Castillo-Lluva, S.; Alvarez-Tabares, I.; Weber, I.; Steinberg, G.; Perez-Martin, J. Sustained cell polarity and virulence in the phytopathogenic fungus *Ustilago maydis* depends on an essential cyclin-dependent kinase from the Cdk5/Pho85 family. *Journal of Cell Science* **2007**, *120*, 1584-1595, doi:10.1242/jcs.005314.
63. Alvarez-Tabares, I.; Perez-Martin, J. Cdk5 kinase regulates the association between adaptor protein Bem1 and GEF Cdc24 in the fungus *Ustilago maydis*. *Journal of Cell Science* **2008**, *121*, 2824-2832, doi:10.1242/jcs.026286.

64. Peleg, Y.; Addison, R.; Aramayo, R.; Metzenberg, R.L. Translocation of *Neurospora crassa* transcription factor NUC-1 into the nucleus is induced by phosphorus limitation. *Fungal Genetics and Biology* **1996**, *20*, 185-191, doi:10.1006/fgbi.1996.0034.
65. Estrada-Rivera, M.; Hernandez-Onate, M.A.; Dautt-Castro, M.; Gallardo-Negrete, J.J.; Rebolledo-Prudencio, O.G.; Uresti-Rivera, E.E.; Arenas-Huertero, C.; Herrera-Estrella, A.; Casas-Flores, S. IPA-1 a putative chromatin remodeler/helicase-related protein of *Trichoderma virens* plays important roles in *Antibiosis* against *Rhizoctonia solani* and induction of *Arabidopsis* systemic disease resistance. *Molecular Plant Microbe Interact* **2020**, *33*, 808-824, doi:10.1094/MPMI-04-19-0092-R.
66. Sun, Z.; Song, J.; Xin, X.; Xie, X.; Zhao, B. Arbuscular mycorrhizal fungal 14-3-3 proteins are involved in arbuscule formation and responses to abiotic stresses during AM symbiosis. *Frontiers in Microbiology* **2018**, *9*, 91, doi:10.3389/fmicb.2018.00091.
67. Oide, S.; Liu, J.; Yun, S.H.; Wu, D.; Michev, A.; Choi, M.Y.; Horwitz, B.A.; Turgeon, B.G. Histidine kinase two-component response regulator proteins regulate reproductive development, virulence, and stress responses of the fungal cereal pathogens *Cochliobolus heterostrophus* and *Gibberella zeae*. *Eukaryot Cell* **2010**, *9*, 1867-1880, doi:10.1128/EC.00150-10.
68. Veneault-Fourrey, C.; Barooah, M.; Egan, M.; Wakley, G.; Talbot, N.J. Autophagic fungal cell death is necessary for infection by the rice blast fungus. *Science* **2006**, *312*, 580-583, doi:10.1126/science.1124550.
69. Josefsen, L.; Droce, A.; Sondergaard, T.E.; Sørensen, J.L.; Bormann, J.; Schäfer, W.; Giese, H.; Olsson, S. Autophagy provides nutrients for nonassimilating fungal structures and is necessary for plant colonization but not for infection in the necrotrophic plant pathogen *Fusarium graminearum*. *Autophagy* **2014**, *8*, 326-337, doi:10.4161/auto.18705.
70. Wang, J.; Xu, C.; Sun, Q.; Xu, J.; Chai, Y.; Berg, G.; Cernava, T.; Ma, Z.; Chen, Y. Post-translational regulation of autophagy is involved in intra-microbiome suppression of fungal pathogens. *Microbiome* **2021**, *9*, 131, doi:10.1186/s40168-021-01077-y.
71. Pham, C.D.; Yu, Z.; Sandrock, B.; Bolker, M.; Gold, S.E.; Perlin, M.H. *Ustilago maydis* Rho1 and 14-3-3 homologues participate in pathways controlling cell separation and cell polarity. *Eukaryot Cell* **2009**, *8*, 977-989, doi:10.1128/EC.00009-09.
72. Paul, J.A.; Barati, M.T.; Cooper, M.; Perlin, M.H. Physical and genetic interaction between ammonium transporters and the signaling protein Rho1 in the plant pathogen *Ustilago maydis*. *Eukaryot Cell* **2014**, *13*, 1328-1336, doi:10.1128/EC.00150-14.
